# Genome-wide association analyses for yield and yield-related traits in bread wheat (*Triticum aestivum* L.) under pre-anthesis combined heat and drought stress in field conditions

**DOI:** 10.1371/journal.pone.0213407

**Published:** 2019-03-18

**Authors:** Mirza Faisal Qaseem, Rahmatullah Qureshi, Humaira Shaheen, Noshin Shafqat

**Affiliations:** 1 Department of Botany, PMAS- Arid Agriculture University, Rawalpindi, Pakistan; 2 Department of Biosciences, COMSATS University, Pakistan; 3 Department of Agriculture, Hazara University Dhodial, Mansehra, Khyber Pakhtunkhwa, Pakistan; Institute of Genetics and Developmental Biology Chinese Academy of Sciences, CHINA

## Abstract

Understanding the genetic basis of heat and drought stress tolerance in wheat is prerequisite for wheat breeding program. In the present study, a wheat panel comprising of 192 elite bread wheat genotypes was phenotyped in eight environments for yield and related traits in field conditions. Four stress environments were created by implying four different treatments differing in sowing date and water availability, panel was evaluated for two years in field conditions. The panel was genotyped with 15K Illumina chip and 9236 polymorphic markers concentrated on B genome were employed in GWAS analysis. Consistent, fast LD decay was observed on D genome and structure analysis germplasm divided panel into three major populations. GWAS was performed using BLUEs values of combined environment data in R package GAPIT using log10(P) = 3.96 as significance threshold. The significance of association was further checked using FDR<0.05 threshold. The GWAS identified 487 loci associated with the traits and were significant at log10(p) threshold out of these 350 loci were significant at FDR threshold. For two stress indices 108 associations were significant at FDR threshold. Nine genomic regions were shared among all treatment, while multiple pleiotropic regions were present on chromosome 7D followed by unmapped chromosome. The present study validated many marker trait associations for yield and other traits, MTAs significant under combined drought and heat stress were novel. These regions are important and can be used for fine mapping and marker assisted selection to discover new genes responsible for heat and drought tolerance in wheat.

## Introduction

Bread wheat is world’s 3rd most cultivated cereal crop planted over more than 20% area and provide 20% calories and 20% plant derived proteins to global population [1,2]. Global wheat demand is increasing with continuous increase in population and it is estimated that there is a need to increase global wheat production by 70% in 2050 [3]. Due to uncertainty in climatic conditions, it is estimated that wheat yield will reduce by 50% in South Asia in 2050 which is 7% of total global crop reduction [4]. This vulnerability in wheat yield is due to changes in patterns of rainfall, increase in temperature and occurrence of simultaneous drought and heat stress during the grain filling period. Increase in air temperature, radiation stress, high levels of CO_2_ and increase in the amount of greenhouse gasses further increase the intensity of drought and heat stress [5–7]. In coming few decade scenario of climate change will worsen, it is predicted that global temperature will increase by 3–5°C and annual precipitation will decrease by 4–27% in different parts of world [8]. The major adverse effects of heat stress on wheat include reduction in crop cycle, increase in soil temperature and rate of evaporation while drought stress mainly effects sink and source strength. Interactive effects of drought and heat stress may come from increased vapour pressure deficit. Many attempts have been made by using conventional breeding strategies to improve grain yield and quality of bread wheat, but these approaches altogether increased yield by less than one percent per year [9–12]. Future wheat breeding program is based on dissecting molecular and genetic basis of heat and drought stress tolerance through complementary approaches of association mapping and QTL mapping [13–15]. Till many years QTL mapping was considered as a powerful tool for genetic dissection of complex traits in plants but now QTL mapping is replaced by association mapping. Association mapping is based on linkage disequilibrium (LD) and is a powerful approach with higher resolution due to presence of higher genetic diversity and historic recombination of alleles among association mapping populations [16]. Association mapping is used to identify genomic regions associated with heat and drought tolerance in many association mapping populations [17–20] but these studies focus only either on heat or drought stress. Only few studies focused on combined drought and heat stress [21–24]. In the present study a diverse panel of bread wheat genotype was phenotyped under optimum [C], drought [D], heat [H] and combined heat and drought stressed [HD] conditions for two cropping years. In addition to assessing genetic diversity of the panel significant markers traits associations were identified for each stress treatment. Furthermore, common association among stress treatment and pleiotropic regions shared by multiple traits were also assessed.

## Materials and methods

### Plant material

The plant material comprised of 192 diverse bread wheat lines from International Wheat and Maize Research Center (CIMMYT) heat and drought nurseries for South Asia. Ten local high yielding varieties were also used in the present study to compare yield and to estimate effects of combination of both stresses on their yield. Detailed information about selection history and entry number is given in S1 Table. Seed of whole germplasm was obtained from Crop Physiology, Laboratory National Agriculture Research Center (NARC), Islamabad. Field was ploughed three weeks prior to seed sowing and fertilizer was added with standard rate. All standard agronomic practices were performed in all plots.

### Experimental setup

**Fig 1:**
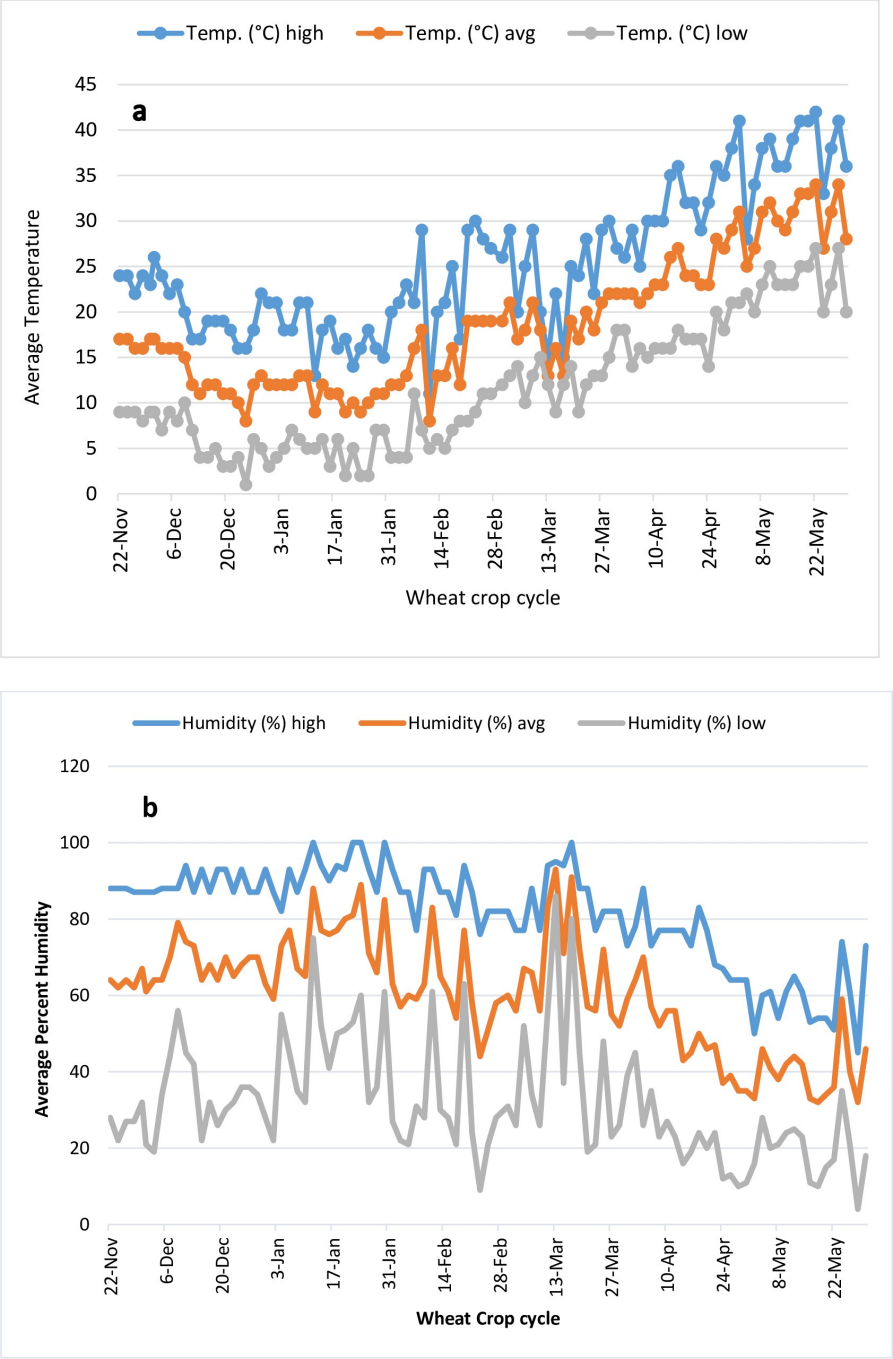
a: Averaged two-year maximum minimum and average temperature of experimental location. b. Averaged two-year maximum, minimum and average percent humidity of experimental location.

The experiments were planted in a lattice design with four replications and net plot size of 3 × 2m. Three rows per genotype with a row length of 2m were sown using a small plot grain drill machine with row to row distance of 22 cm for two cropping seasons. The averaged value of minimum, maximum and average percent humidity and temperature for both cropping seasons are given in Fig 1A and Fig 1B. Following stress treatments were imposed on entire germplasm for two years. The Control [C] or non-stress treatment genotypes were sown at 22 November and grown at 100 percent moisture content throughout cropping cycle. For drought [D] treatment genotypes were sown at 20 November and grown at 35% field capacity after heading. For Heat [H] stress the planting date was 01 January and germplasm was grown at 100% field capacity after heading. For a combination of heat and drought stress [HD] treatment germplasm was sown at 01 January and grown at 35% field capacity after heading. The germplasm in all plots was grown with normal agronomic practices and 100% field capacity till 50% heading S1 Fig. The treatments were started after heading and last till maturity after which all the plots were rehydrated. The [HD] and [D] treatment were protected from the rain after heading by covering tunnel with polythene sheaths and to avoid percolation of water one-meter-deep ditch was dug around the boundary and polythene sheet was inserted in it to avoid rain water seepage inside the tunnel.

## Data recording

The yield traits were recorded from all three plants per genotype while physiological data was recorded from three randomly selected plants. Following yield traits were recorded during study; awn length (AL) (from tip of spike), days to anthesis (DTA) (number of days taken from emergence to appearance of anthers), day to maturity (DTM) (number of days taken from emergence to maturity), grains per spike (GPS) (number of grains in spike was counted), grain yield (GY) (weighing grains form all harvested plants), harvest index (HI) (percent ratio of grain yield and above ground plant dry weight (DW), peduncle extrusion (PEXT) (from tip of flag leaf to base of spike), peduncle length (PL) (from first node to base of spike), plant height (PH) (from ground level to spike tip excluding awns), spikelet number (SLP) (counting number of fertile tillers), spike length (SL) (manually with ruler in cm). Stress tolerance index (STI) Tolerance index (TOL)

$$Stress\;Tolerance\;Index=\frac{Yp-Ys}{{Yp}^{2}}$$

Toleranceindex=Yp−Ys

where Ys and Yp represent yield under stress and non-stress treatment and Yp^2^ is the mean yield of wheat lines evaluated under non-stress conditions [25].

### Statistical analysis

Summary statistics, Pearson’s correlation analyses, broad sense heritability estimates and association mapping were estimated from best linear unbiased estimated values calculated from mean value of each trait keeping genotype as fixed factor in Genestat v 18. The germplasm was genotyped with 15K Illumina chip and filtering of polymorphic markers was done following [26]. Map position of SNPs was indicated by using wheat genetic map published by [27], after filtering 9236 polymorphic markers were used in GWAS analyses. Genetic diversity, polymorphic information content and major allele frequency was estimated by using Powermarker v3.25 (http://statgen.ncsu.edu/powermarker) software. GenStat v18 was used to calculate chromosome-wise linkage disequilibrium (LD) decay by calculating pairwise marker allele squared correlation (r^2^) and plotting the r^2^ values against the genetic distances (cM). Population structure was estimated using STRUCTURE v 2.3.4 [28], as described by [29]. All the polymorphic markers were used to determine population structure by inferring K from 2 to 10 using 100,000 burn-in iterations followed by 100,000 MCMC (Markov-Chain Monte Carlo) iterations and 5 replications for each K. The obtained results were analyzed using STRUCTURE harvester (http://taylor0.biology.ucla.edu/structureHarvester) to get appropriate K value.

### Association mapping

Best linear unbiased estimate values of each trait were used to perform GWAS using mixed linear model approach [30,31] in R package GAPIT (Genome Association and Prediction Integrated Tool) [32]. The correction for population stratification and cryptic relatedness was performed by employing coefficient of co-ancestry kinship and first three principal components as random effects in the linear mixed-effect model [33]. Appropriateness of model used for association analysis in present study was checked by drawing QQ plots between expected and observed log10(P) values. Significance threshold was set by following [34] which was equal or greater than log10 (P) ≥3.98 and after application of Bonferroni correction the log10 (P) threshold rose to 5.26 [35].

## Results

### Phenotypic results

Grain yield varied greatly across the environments, high grain yield was recorded under non stress treatment [C] (Average = 387.24, H^2^ = 0.86) followed by Heat [H] (Average = 281.40, H^2^ = 0.86), drought [D] (Average = 290.80, H^2^ = 0.97) and combined heat and drought treatment [HD] (Average = 187.16, H^2^ = 0.83). These results showed that independent heat had more pronounced effects on grain yield than independent drought stress while combination of both stresses had more severe effects than independent drought or heat stress. The same trend was observed for other traits and FLL, FLW HI, LA and TILL were more effected by drought stress than heat and regarded as drought sensitive traits while DW, DTM, PH, SL and SPLS were more effected by heat stress and regarded as heat sensitive traits. The heritability values were higher under all stress treatment with DW and GY having highest H^2^ value under [C] treatment, FLL and LA had highest H^2^ value under [D] treatment, FLL had highest H^2^ value under [H] treatment and DTM had highest H^2^ value under [HD] treatment. The range, mean and heritability values for yield and related traits in the normal environment [C], drought [D], heat [H] and combined heat and drought [HD] stressed environments are summarized in Table 1.

**Table 1:** Averages and standard deviations for eleven traits evaluated in the GWAS in four stressed environments under control [C], drought [D], heat [H] and combined drought and heat [HD] stress for two cropping seasons.

	CONTROL				DROUGHT				HEAT				HEAT*DROUGHT			
Trait	Min	Max	Mean	H2	Min	Max	Mean	H2	Min	Max	Mean	H2	Min	Max	Mean	H2
DW	246.5	878.49	542.94	0.96	186.72	715	459.43	0.83	218	653.14	419.5	0.76	150.5	618	372.99	0.96
DTM	132	160	145.5	0.88	121	147	133.23	0.64	109	138.5	121.91	0.9	104.9	124.02	117.09	0.97
FLL	23.03	44.4	32.95	0.96	16.89	39.09	26.62	0.98	20	40.7	30.77	0.99	14.77	32.1	22.37	0.92
FLW	1.46	3.35	2.41	0.92	1.29	2.64	1.9	0.8	1.47	3.16	2.17	0.93	1.02	2.65	1.69	0.77
GY	216.5	565.5	387.24	0.86	108.5	490.5	290.8	0.97	212.3	360	281.4	0.86	102	286.5	187.16	0.83
HI	40.08	88.49	59.51	0.95	30.17	70.92	48.62	0.96	33.77	71.94	52.94	0.92	16.62	61.32	38.5	0.59
LA	31.57	73.03	50.8	0.94	22	65.67	40.09	0.98	32.16	65.85	46.09	0.86	20	44.46	30.87	0.69
PH	66.83	109.67	86.84	0.61	61.13	104.13	80.34	0.5	59.33	85.64	71.31	0.79	44	75.77	60.63	0.89
SL	11.17	20.63	16.48	0.91	9	18.67	14.26	0.85	8.67	19.37	13.67	0.79	6.68	14	10.2	0.79
SPLS	13.33	24.67	19.56	0.94	12.01	22.96	17.86	0.97	9.5	19.83	14.46	0.92	8.17	15.96	11.36	0.96
TILL	4	11	7.27	0.39	4	9.47	6.27	0.52	3.88	9.14	6.43	0.77	1.33	7.3	4.82	0.53

DW, Plant Above Ground Dry Weight; DTM, Days to Maturity; FLL, Flag Leaf Length; FLW, Flag Leaf Width; GY, Grain Yield; HI, Harvest Index; LA, Leaf Area; PH, Plant Height; SL, Spike Length; SPLS, Spikelets per spike; TILL, Tillers per plant

Min, Minimum value; Max, Maximum value, H2, Broad sense heritability

**Fig 2:**
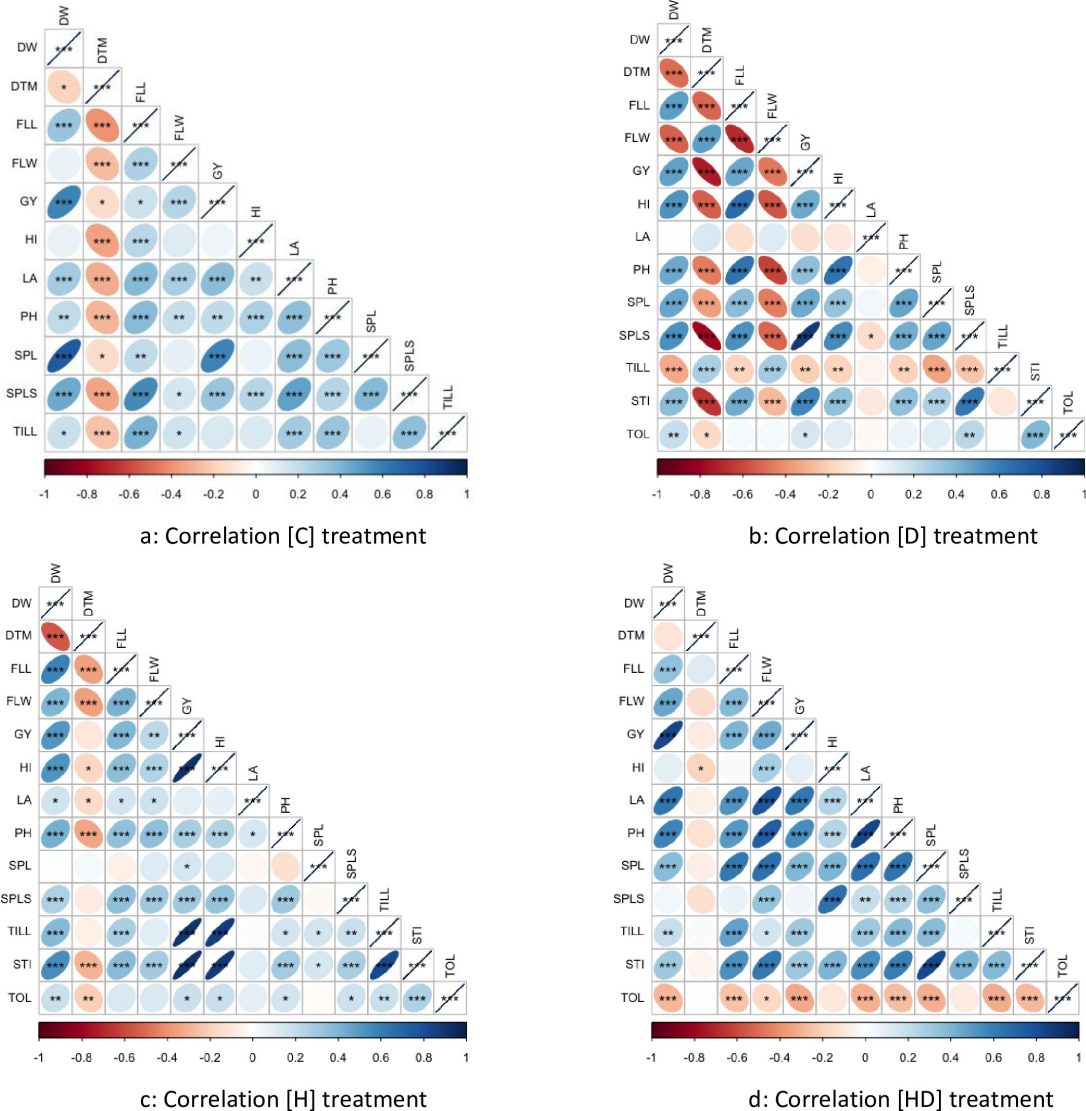
Pearson correlation between grain yield and all studied traits and stress indices. **a:** Correlation under non stress treatment **b:** correlation under drought stress **c:** correlation under heat stress **d:** correlation under combined heat and drought stress.

Grain yield showed consistent negative correlation with day to maturity under all stress treatments, suggesting that early maturing plants had higher yield than the late maturing plants under stressed environment. Under non stress treatment [C] grain yield had highest positive correlation with plant above ground dry weight (r = 0.58**) while under drought stress [D] it had highest positive correlation with Spikelets per spike (r = 0.88**), under heat stress it had highest correlation with harvest index (r = 0.92**) and under combined heat and drought stress it had highest correlation with plant above ground dry weight (r = 0.83**). Stress tolerance index had positive correlation with grain yield under all stress treatments while tolerance index had positive correlation under individual drought (r = 0.14*) and individual heat stress (r = 0.18*) but it had negative correlation with grain yield under combined drought and heat stress (r = -0.33**). The detailed information about correlations among all studied traits is given in Fig 2. The results from analysis of variance showed that genotype, treatment, cropping years and their interaction had significant effects on studied traits.

### Genotypic results

**Fig 3:**
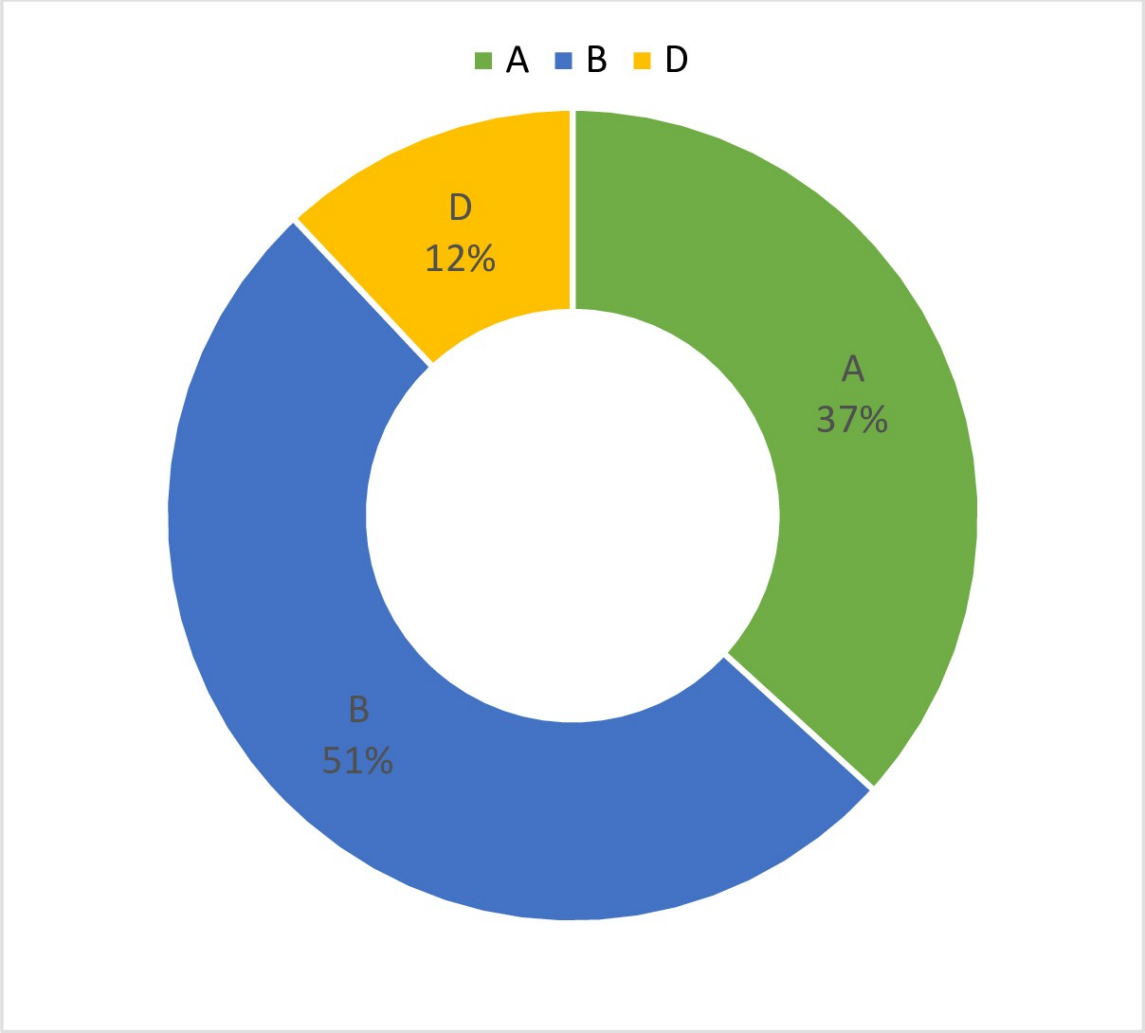
Distribution of polymorphic SNP markers on different wheat genomes.

The association mapping panel was mapped with 15k ilumina chip from Gatersleben, Germany. After filtering (removing SNPs with MAF <5%), 9236 polymorphic markers were employed in GWAS analysis. Most of these polymorphic SNPs were concentrated on B genome (51%) followed by A (37%) and D (12%) genome (Fig 3). Chromosome 2B had highest number (903) of polymorphic SNPs while chromosome 4D had least number (42) of these SNPs. The average polymorphic information content was 0.27 with maximum PIC value (0.35) for chromosome 6B and least (0.14) for chromosome 5D (S2 Fig). Based on R^2^ model highest LD decay (r^2^ = 0.64) was seen on chromosome 4D while least LD (r^2^ = 0.38) was seen on chromosome 5A with an average Pearson's correlation (r^2^) value of 0.45. The LD decay was faster for A and B genome resulting smaller genetic distance when compared to D genome. The rate of LD decay was ~8 cM; ~5 cM ~10 cM for A, B and D genome, respectively with mean genetic length of 8.1 cM for all groups showing that one marker is enough within ~8.1 cM region for each chromosome (S1 Appendix).

### Population structure

**Fig 4:**
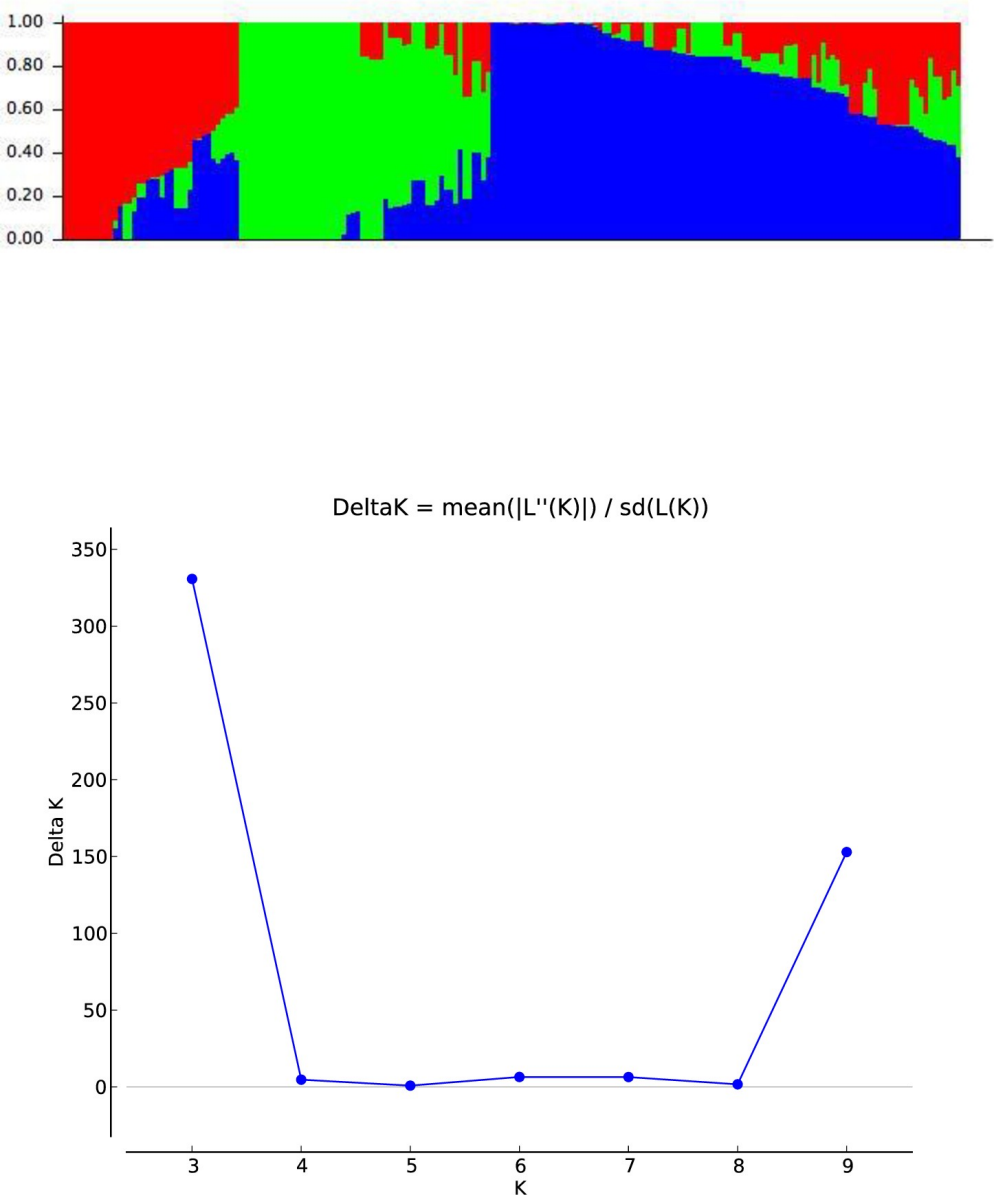
STRUCTURE analysis used to define genetic relationships in the AM panel. a) Each horizontal entry represents one AM panel entry. The existence of three sub-populations was inferred. b) STRUCTURE analysis used to define genetic relationships in the AM panel. Δk plot, with k ranging from1-10.

Population structure analysis divided the whole population in three sub-populations, population_01 containing 36 genotypes while population_02 and population_03 contained 51 and 105 genotypes respectively. The members of population _01 contained genotypes with VORB, PASTOR and PWB65 as one of their parent while genotypes of population_02 contained CHIBIA, KACHU #, ND643 and PRL as one of their parents. The population_03 contained maximum 54% genotypes of whole studied panel and most of these genotypes contained ATTILA, KIRITATI, PASTOR, SERI and WAXWING as one of their parents (Fig 4A and 4B, S1 Table).

### Association mapping

**Fig 5:**
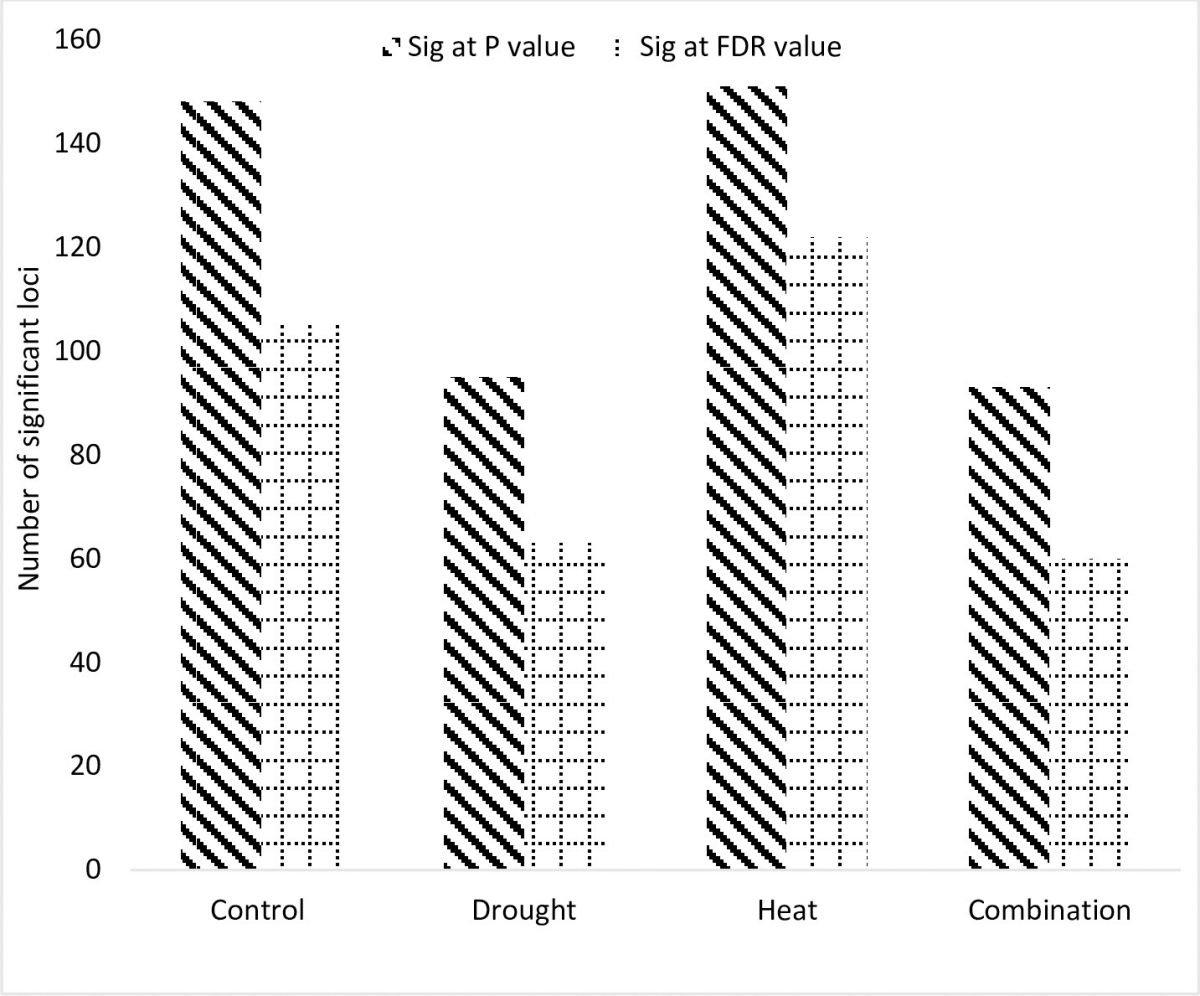
Number of loci significantly associated with studied trait under [C], [D], [H] and [HD] treatments.

Genome wide association mapping was performed for independent drought and independent heat stress and for combination of both heat and drought stress using R package GAPIT with a significance threshold of log 10(P)>3.98 and FDR<0.05. Total 148, 95, 151 and 93 significant associations were detected for studied traits under non stress [C], drought [D], Heat [H] and for combined heat and drought [HD] treatment respectively. Out of these, 105, 63, 122 and 60 were significant at FDR threshold under non stress [C], drought [D], Heat [H] and for combined heat and drought [HD] treatment respectively (Fig 5). Manhattan and QQ plots for each trait under each stress treatment are given in (S2 Appendix).

#### Marker trait association under non-stress treatment [C]

Under optimum growing conditions 148 marker trait associations (MTAs) were significant at Log(P) value threshold while 105 were significant at FDR threshold. Maximum number of significant associations (36) were reported for plant above ground biomass (DW) followed by 23 significant associations for grain yield (GY). Only one marker significant at Log(P) threshold was associated with leaf area under [C] treatment while no significant association was recorded at FDR threshold for FLL, HI, LA and PH. On average all significant associations under [C] treatment explained 20.25% phenotypic variation. Maximum number of associations (18) were recorded on chromosome 3D followed by 17 associations on 5B and 6A while chromosome 2D and 3A had only one significant association. Many genomic regions on chromosome 3D were pleiotropic and were associated with more than one trait. The most potent MTAs for grain yield under optimum conditions [C] were recorded on chromosome 3D (GENE-1752_162 at 101.24 and RFL_Contig2471_119 at 23.41 cM) and explained 28.65 and 23.61% phenotypic variation. Grain yield had common QTL with DW, DTM, FLL, FLW, HI, SPLS and TILL and most consistent regions controlling these traits were present on 3D, 5B and 6A (Table 2, S2 Table).

**Table 2:** Centi-Morgan position of significant marker traits associations detected for yield and related traits from BLUEs value under non stress [C] treatment.

Chromosome	DW	DTM	FLL	FLW	GY	HI	PH	SPL	SPLS	TILL
**1A**	51.09, 71.05	-	-	-	-	-	93.61	-	-	-
**1B**	30.68, 43.86, 62.58, 63.8	-	-	158.59	158.59	158.59	-	-	158.59	158.59
**1D**	87.36, 88.85, 71.47	-	-	-	-	-	-	-	-	-
**2B**	-	-	48.54	-	96.14	-	108.04	-	96.14	96.14
**2D**	97.14	-	-	-	-	-	-	-	-	-
**3A**	26.01,87.78, 86.16,97.14	-	-	-	-	-	-	-	-	-
**3B**	37.29, 67.67, 67.78, 137.84	-	-	-	-	-	-	-	-	-
**3D**	101.24	23.41,101.24	23.41, 25.11	142.32, 143.01	23.41, 101.24	-	-	-	23.41,101.24	23.41, 101.24, 82.56, 96.33, 97.66
**4A**	-	-	-	48.84	-	-	-	-	-	-
**4B**	5.99	-	-	-	-	-	34.15	-	-	-
**5A**	-	-	-	-	53.47	-	-	-	-	-
**5B**	11.23	32.73, 49.01, 100.64, 141.91	32.73	141.91	32.73, 49.01, 100.64, 141.91	141.91	-	109.53, 115.69	32.73	32.73
**5D**	-	69.96	-	-	69.96	-	-	-	-	-
**6A**	85.07	133.74	-	133.74	133.74	133.74	140.7	25.53, 27.64, 37.03, 40.6, 43.1	-	-
**6B**	-	-	-	-	-	-	-	67.24, 71.76	-	-
**6D**	153.08	-	-	-	153.08	-	-	-	-	-
**7A**	-	63.21	-	-	63.21	-	-	-	-	-
**7B**	49.38,61.14	-	-	89.82, 90.42, 92.52, 143.23, 144.8, 148.65, 150.6	163.3	148.65	-	-	163.3	163.3
**7D**	197.58, 203.58	-	-	-	208.1	-	-	-	208.1	208.1

DW, Plant Above Ground Dry Weight; DTM, Days to Maturity; FLL, Flag Leaf Length; FLW, Flag Leaf Width; GY, Grain Yield; HI, Harvest Index; LA, Leaf Area; PH, Plant Height; SPL, Spike Length; SPLS, Spikelets per spike; TILL, Tillers per plant

#### Marker trait association under drought stress treatment [D]

Under Drought stress treatment [D], 95 associations were significant at Log(P) threshold while 63 associations were significant at FDR threshold. The maximum number of associations (22) were recorded for TILL followed by STI (19) and only a single marker was significantly associated with leaf area. Tillers per plant and stress tolerance index had also highest number of significant associations at FDR threshold each with 10 and 19 associations respectively. Harvest index, leaf area and spike length had no significant association beyond FDR threshold. Under [D] treatment, all the significant associations explained 19.33% phenotypic variation with maximum phenotypic variation (28.93% each) explained by markers IACX203 (67.24 cM) and wsnp_Ex_c18372_27196625 (71.76 cM) on chromosome 5D and associated with STI. Chromosome wise highest number of significant associations (24) were detected on chromosome 7B followed by 17 associations on chromosome 2B.

Significant associations for GY under [D] stress were detected on chromosome 3D, 6A, 6D, 7B and on unmapped chromosome, CAP7_c1274_206 on unmapped chromosome was most significantly associated with GY and explained 23.49% phenotypic variance. Pleiotropic regions under [D] stress were concentrated on chromosome 7B followed by chromosome 4B, chromosomal region significant for GY on 7B was ~15 cM apart from region associated with DW, FLW, HI, LA, PH and TILL. The chromosomal region on 3D, 6A and 6D were specifically associated with grain yield (Table 3, S2 Table).

**Table 3:** Marker Centi-Morgan position of significant marker traits associations detected for yield and related traits from BLUEs value under drought stress [D] treatment.

Chromosome	DW	DTM	FLL	FLW	GY	HI	LA	PH	SPL	SPLS	TILL
**1B**	-	-	-	-	-	-	-	-	-	-	56.88
**2B**	-	-	-	-	-	-	-	-	-	109.53	109.53
**3D**	-	-	-	-	66.57	-	-	-	-	-	-
**4B**	-	55.55, 55.96	-	55.55, 55.96	-	-	-	-	55.96	55.55, 55.96	55.55, 55.96, 62.92
**6A**	-	-	-	28.46, 29.53, 31.89	6.98	-	-	-	-	-	-
**6B**	-	-	-	-	-	-	-	-	0.37	-	-
**6D**	-	-	-	-	9.47	-	-	-	-	-	-
**7A**	-	178.42	-	-	-	-	135.81	-	-	-	-
**7B**	163.3	-	163.3	-	134.06, 148.65, 150.6	163.3	163.3	163.3	-	-	76.31, 73.79, 71.66, 163.3, 171.11
**7D**	208.1	-	208.1	-	-	208.1	208.1	208.1	-	-	-

#### Marker trait association under heat stress treatment [H]

Under [H], 151 associations were significant at Log(P) threshold while 122 of these were significant at FDR. TOL had maximum number (21) of significant associations followed by STI (20) and FLL (12) and all these MTAs were also significant at FDR. Only DW had no significant association at FDR threshold among all the studied traits. Under [H] stress the potent associations for grain yield were present on two chromosomes viz. 7B and unmapped chromosome and IACX5767 on 7B explained 21.62% phenotypic variation. All significant associations under [H] stress explained 20% phenotypic variation with maximum value of phenotypic variation of 24.17% for leaf area. Grain yield under [H] stress shared genomic region with all studied traits except FLL, PH, SL and TILL and these common genomic regions were present between 134.06–150.6 cM on chromosome 7B (Table 4, S2 Table).

**Table 4:** Centi-Morgan position of significant marker traits associations detected for yield and related traits from BLUEs value under heat stress [H] treatment.

Chromosome	Biomass	DTM	FLL	FLW	GY	HI	LA	PH	SPL	SPLS	TILL
**2A**	-	-	52.74	-	-	-	-	52.74, 53.18	-	-	-
**3A**	-	-	89.04, 89.48	-	-	-	-	89.48	-	-	-
**3D**	66.57	-	-	-	-	-	66.57, 142.32	-	-	-	-
**5B**	-	-	-	-	-	-	129.83	-	-	-	-
**6A**	-	6.98	-	6.98	-	-	-	-	-	6.98	-
**6D**	-	9.47	-	9.47	-	-	-	-	-	9.47	-
**7A**	-	178.42	-	178.42	-	-	-	-	-	178.42	-
**7B**	134.06, 148.65, 150.6	134.06, 148.65, 150.6	-	134.06, 148.65, 150.6	134.06, 148.65, 150.6	134.06, 143.23, 144.8, 148.65, 150.6	134.06, 148.65, 150.6	-	-	134.06, 148.65, 150.6	-
**7D**	-	-	149.97, 197.58, 202.54, 206.75	-	-	-	-	149.97, 197.58, 202.54, 206.75	161.13, 197.58, 202.54, 203.58	-	161.13, 197.58, 202.54, 203.58

#### Marker trait association under combined heat and drought stress treatment [HD]

Under [HD] treatment, 93 associations were significant at Log(P) threshold while 60 of these were significant at FDR. Maximum numbers of significant associations were recorded for STI (29) and TOL (13) index and both these traits have higher number of significant associations on FDR threshold. DTM, FLW, GY and TILL were only agronomic traits which had association significant at FDR threshold. Chromosome wise, highest associations were recorded on chromosome 4B (12) followed by 2B, 7B and 7D each carrying 10 significant MTAs. MTAs for grain yield were present on 1A, 2D, 2B, 4A, 7B, 7D and on unmapped chromosome, the potent marker Kukri_rep_c68068_95 was present on chromosome 2D and explained 22.55% phenotypic variation. All significant associations significant for all studied traits on average explained 19% phenotypic variation with maximum contribution (23.67%) by RFL_Contig854_2253 present on 3A significantly associated with TOL. Grain yield share many pleotropic regions with other traits and most of these pleiotropic regions were concentrated on chromosome 7B and 7D where shares the chromosomal region with FLL, FLW, HI, PH, SL, SPLS and TILL (Table 5, S2 Table).

**Table 5:** Centi-Morgan position of significant marker traits associations detected for yield and related traits from BLUEs value under Combined drought and heat stress [HD] treatment.

Chromosome	Biomass	DTM	FLL	FLW	GY	HI	LA	PH	SPL	SPLS	TILL
**1A**	-	-	-	108.06	108.06	-	-	-	-	-	-
**2B**	-	-	-	16.88	16.88	-	-	-	-	-	-
**2D**	-	-	-	100.19	100.19	-	-	-	-	-	-
**4A**	-	-	-	48.52	48.52	-	-	-	-	-	-
**4B**	-	61.31, 61.84, 62.56, 62.92	-	-	-	-	-	55.55, 55.96	-	55.55, 55.96	-
**5A**	15.61	-	-	-	-	-	-	-	-	-	-
**5B**	60.31	-	-	-	-	-	40.55, 43.56, 45.36, 45.4, 46.47	-	-	-	-
**5D**	-	69.13	-	-	-	-	-	135.81	-	-	-
**7A**	-	-	-	-	-	-	-	-	-	-	-
**7B**	-	-	163.3	98.3, 163.3	98.3, 163.3	163.3	-	163.3	163.3	163.3	163.3
**7D**	-	-	208.1	208.1	208.1	208.1	-	208.1	208.1	208.1	208.1

#### Marker trait association for drought stress indices [D]

Marker trait associations for STI under [D] stress were recorded on chromosome 1A, 1B, 1D, 2B, 3A, 3D and 5D. BS00077789_51 on chromosome 2B at 37.03 was most potent loci associated with STI under drought stress and explained 29.08% phenotypic variation. Many genomic regions were shared between STI and other stress indices for example, genomic region on chromosome 2B and 5D were shared by STI and TOL under [D] treatment. Genomic regions significant for TOL under [D] stress were detected on chromosome 2B, 4D and 5D with the highest number of associations on chromosome 5D. The genomic region significantly associated for TOL under drought stress was common for both STI and TOL under all stress treatments. Locus BS00077789_51 on 2B at 37.03 cM was most significantly associated with TOL and explained 26.69% phenotypic variation (Table 6, S2 Table).

**Table 6:** Centi-Morgan position of significant marker traits associations detected for stress Indices for grain yield under all studied stress treatments.

Chromosome	STI_D	STI_H	STI_HD	TOL_D	TOL_H	TOL_HD
**1A**	60.25, 80.13	51.07, 60.25, 61.41	60.25, 61.89, 69.53	-	25.09, 76.6, 80.13	-
**1B**	31.10	-	-	-	29.6, 31.1	28.1, 29.6, 31.1, 37, 37.7
**1D**	132.74	-	-	-	-	134.75
**2B**	25.53, 27.64, 37.03, 40.6, 43.1	37.03, 40.6	37.03, 40.6, 85.07	25.53, 27.64, 37.03, 37.13, 40.6, 43.1	25.53, 27.64, 37.03, 37.13, 40.6, 43.1	25.53, 27.64, 37.13, 43.1
**3A**	81.91, 209.25	-	51.36	-	81.91, 209.25	81.91
**3D**	11.23, 176.18	11.23, 94.89, 96.7, 97.28, 127.96, 141.91, 176.18	11.23, 96.7, 97.28, 110.2, 141.91, 176.18	-	176.18	-
**4A**	-	-	67.72,71.47	-	-	-
**4B**	-	42.91	99.79	-	-	-
**4D**	-	97.71	31.04, 40.94,97.71	158.59	-	-
**5A**	-	-	87.78	-	-	-
**5D**	25.45, 25.82, 67.24,71.76	67.24, 71.76	39.24, 67.24, 71.76	67.24, 71.76	25.45, 25.82, 67.24, 71.76	69.56
**6A**	-	4.56	-	-	146.51	-
**6B**	-	121.6	10.06	-	-	-

STI_D: Stress Tolerance index for GY under drought stress

STI_H: Stress Tolerance index for GY under heat stress

STI_HD: Stress Tolerance index for GY under combined drought and heat stress

TOL_D: Tolerance index for GY under drought stress

TOL_H: Tolerance index for GY under heat stress

TOL_HD: Tolerance index for GY under combined drought and heat stress

#### Marker trait association for heat stress indices [H]

Twenty significant MTAs were recorded for STI under [H] stress and were concentrated on chromosome 3D. The most significant association was present on 3D at 11.23 cM and explained 22.56% phenotypic variation. The region on 3D at 176.18 cM was also significant for TOL index. The TOL index had 21 significant associations under [H] stress most of which were concentrated on chromosome 2B and on average explained 21% phenotypic variation. The most potent association on 2B was present at 37.03 cM and explained 28.8% phenotypic variation. TOL index shared regions on chromosome 2B with stress indices under studied [D], [H] and [HD], similarly genomic region significant at chromosome 5D was also shared among all stress indices in all three treatments (Table 6, S2 Table).

#### Marker trait association for combined heat and drought stress indices [HD]

Twenty-nine significant associations were detected for STI under [HD] treatment concentrated on chromosome 3D and explaining average 19.62% phenotypic variation. IACX203 (67.24 cM) and wsnp_Ex_c18372_27196625 (71.76 cM) were most potent loci associated with STI under [HD] treatment and explained 21.22% phenotypic variation. Thirteen significant associations were detected for TOL under [HD] treatment concentrated on 2B and average phenotypic variation value of 20.05%. RFL_Contig854_2253 on 3A at 81.91 cM was most significant loci for TOL and explained 23.67% phenotypic variation (Table 6, S2 Table).

### Comparison between different stress treatments

**Fig 6:**
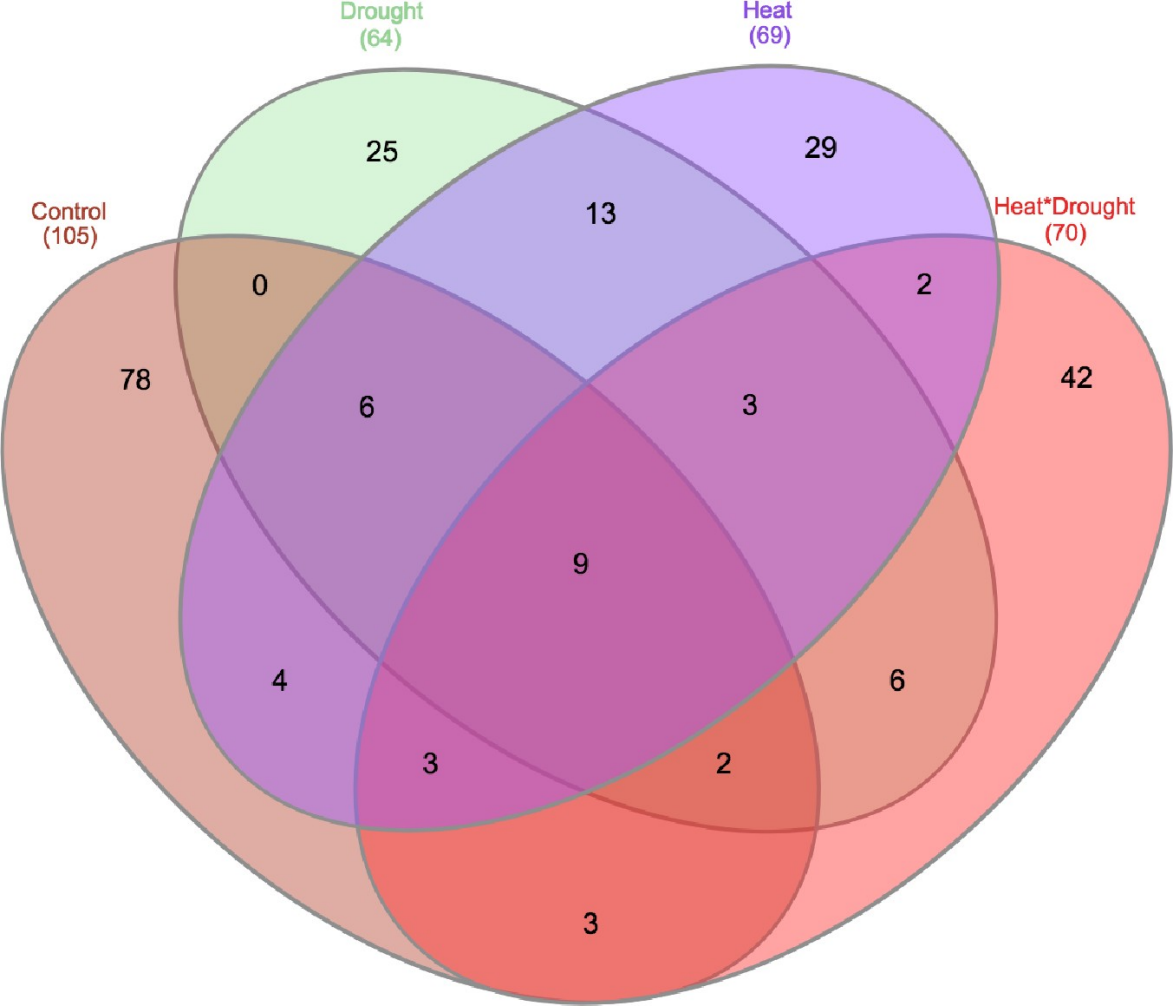
Stress specific and consistent associations shared among more than one stress treatments.

Many significant associations were shared between two or more stress treatments. [C] and [H] stress share four loci, [D] and [H] share 13 loci, [C] and [HD] share 3 loci, [H] and [HD] had two common loci while no common locus was recorded between [C] and [D] treatment. Some loci were common among the three treatments were also recorded [C], [D] and [H] had six loci in common [C], [H] and [HD] had three loci common among them [C], [D] and [HD] had two loci in common and [D], [H] and [HD] had three loci in common. Nine loci were common among all four treatments and were recorded as consistent loci (Fig 6).

### Traits having common associations

**Fig 7:**
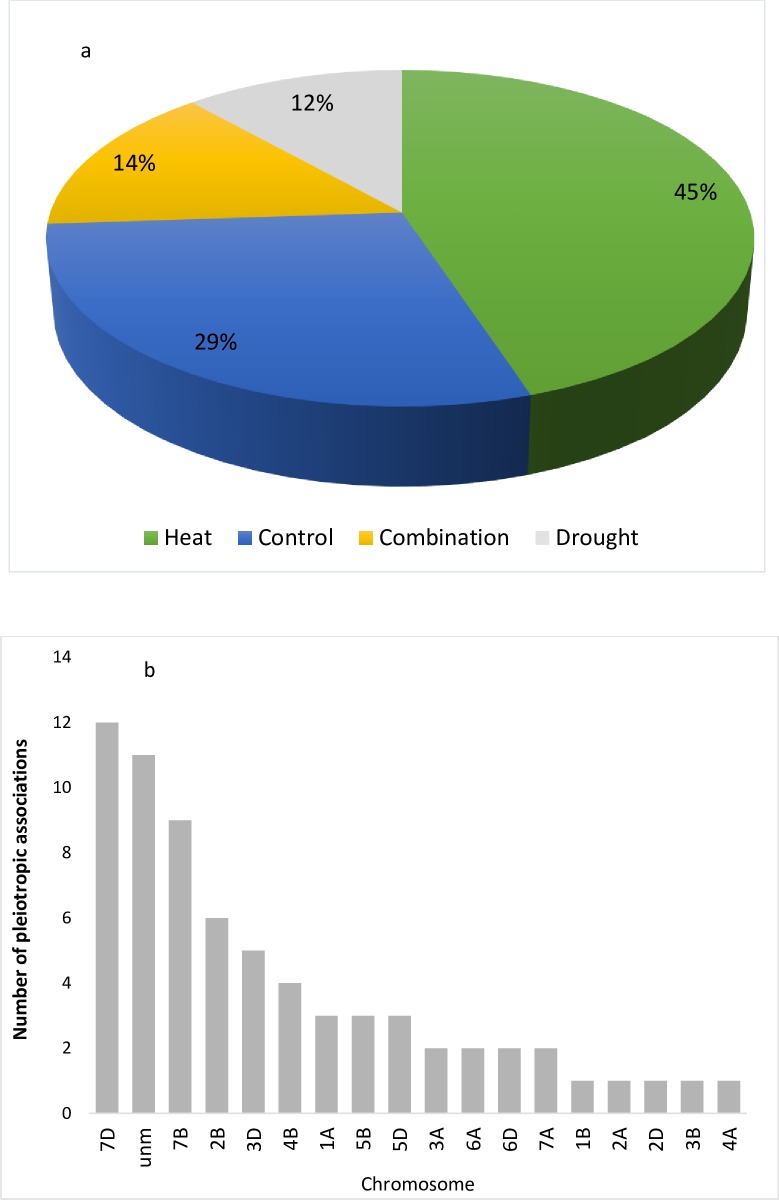
a) Number of peliotropic SNPs associated with studied traits under all stress treatments i.e. [C], [D], [H] and [HD]. b) Chromosome wise distribution of pleiotropic SNPs associated with traits under all stress treatments.

A total 69 pleiotropic SNPs were associated with multiple traits under all stress treatments, [H] with maximum (31) of these followed by [C], [HD] and [D] each having 20, 10 and 8 associations respectively (Fig 7A). The maximum number of pleiotropic associations were recorded on chromosome 7D followed by unmapped chromosome and 7B (Fig 7B). Locus BS00022775_51 was associated with eight studied traits, i.e. Biomass, DTM, FLW, GY, HI, LA, SLPS and SL, similarly locus BobWhite_c28058_232 and wsnp_Ex_c8400_14157060 on chromosome 7B at 134.06 cM were associated with seven out of eleven studied traits. Under [C] treatment locus Tdurum_contig52086_129 on chromosome 1B at 158.59 cM was associated with six multiple traits while BobWhite_s64797_152 and Tdurum_contig33737_157 on 4B at 55 cM were associated with five studied traits under [D] stress. Locus BobWhite_c28058_232 and wsnp_Ex_c8400_14157060 on chromosome 7B at 134.06 cM were associated with seven traits under [H] stress while locus RAC875_rep_c110526_324 on 7B at 163.3 cM was associated with 8 studied traits under [HD] treatment (Table 7).

**Table 7:** Chromosome, centi Morgan position and associated traits of pleiotropic SNPs recoded from association analysis of BLUEs values.

Significant SNPs	Chr.	Position	Control [C]	Drought [D]	Heat [H]	Combination [HD]
wsnp_Ex_c33831_42253707, wsnp_Ku_c17726_26872129	1A	70.1–71.05	Biomass, DTM			
Excalibur_c26688_138	1A	108.06				FLW, GY
Tdurum_contig52086_129	1B	158.59	DTM, FLL, GY, HI, SPLS, TILL			
RAC875_c62936_139	2A	52.74			FLL, PH	
Excalibur_c2656_3198	2B	16.88				FLW, GY
BS00077789_51, tplb0060j17_879	2B	37.03–40.6			STI, TOL	
IAAV1903	2B	96.14	GY, SPLS, TILL			
Excalibur_c6922_1393-Kukri_s115194_71	2B	109.53		SPLSS, TILL		
Kukri_rep_c68068_95	2D	100.19				FLW, GY
wsnp_Ex_c14340_22315611, wsnp_Ex_rep_c66907_65324299	3A	89.48			FLL, PH	
Tdurum_contig100787_79	3B	137.84	Biomass, SPL			
RFL_Contig2471_119	3D	23.41	DTM, FLL, GY, SPLS, TILL			
BS00021930_51	3D	66.57			Biomass, LA	
BS00095640_51	3D	82.56	SPLS, TILL			
GENE-1752_162	3D	101.24	Biomass, DTM, GY, SPLS, TILL			
BS00029720_51	3D	176.18			STI, TOL	
Ex_c17894_1159	4A	48.52				FLW, GY
BobWhite_s64797_152, Tdurum_contig33737_157	4B	55.55–55.96		DTM, FLW, SPL, SPLSS, TILL		PH, SPLS
Tdurum_contig49841_618	5B	32.73	DTM, FLL, GY, SPLS, TILL			
Ra_c22730_460	5B	100.64	DTM, GY			
BS00029540_51	5B	141.91	DTM, FLW, GY, HI			
IACX203, wsnp_Ex_c65985_64188864, wsnp_Ex_c18372_27196625	5D	67.24–71.76	DTM, GY		STI, TOL	
Tdurum_contig27939_357	6A	6.98			DTM, FLW, SLPS	
BobWhite_rep_c63956_254	6A	133.74			DTM, FLW, GY, HI	
Excalibur_c4789_2748	6D	9.47			DTM, FLW, SLPS	
RFL_Contig2615_982	6D	153.08	Biomass, GY			
Kukri_c34887_734	7A	63.21	DTM, GY			
wsnp_Ex_c53442_56678505	7A	178.42			DTM, FLW, SLPS	

## Discussion

In last few decades lots of efforts had been made to study the effects of a single stress on plants under control and field conditions [36]. However, in open field conditions, plant have to face multiple biotic and abiotic stresses at same time and one cannot really understand the effects of these combined stresses by applying one stress separately in controlled lab conditions. The combination of two or more stresses alters plant proteomics, metabolism and genetics in a unique way which is totally different from changes imposed by individual stress [23,37,38]. To address this scenario, we aimed to determine independent and combined effect of drought and heat stress on wheat yield and related traits and finding out genomic regions associated with heat and drought stress tolerance.

A diverse panel of 192 advance wheat lines was planted at National Agriculture Research Center Islamabad for two cropping seasons in four treatments, i.e. [C], [D], [H] and [HD]. The GY was severely reduced by [HD] stress (51.67% reduction) followed by [H] (27.33% reduction) and [D] treatment (24.9% reduction). Similar results were reported by [15,39,40] for bread wheat genotypes grown under heat and drought stressed environments. Heritability estimates were high for many traits showing uniformity in the performance of genotypes across the years, these values are similar as reported by [26,41]. Consistent negative association of GY with DTM under stressed conditions suggests early maturity is beneficial in stressed environments [42]. Plant height had a consistent positive association with grain yield this might be due to positive correlation of PH with DW [43–45]. This suggests that under stressed environment tall genotypes can accumulate and mobilize more reserve to grain and thus had higher yield than short stature genotypes [43].

Association mapping is a powerful approach to elucidate genetic basis of complex traits including grain yield [41,46–48], in spite of its high-resolution there are higher chances to detect false positive associations due to population structure [49,50]. In our study mixed linear model was used to detect significant MTAs and significance was further tested to avoid false positive associations using FDR threshold. The MTAs for GY were detected on almost all chromosomes with maximum number of significant associations under [C] treatment. QTLs for grain yield and its related traits are reported on almost all wheat chromosome [51–54]. The maximum number of associations for grain yield were detected on chromosome 7B under both stress and non-stress conditions. The association on 7B at 134.06 cM was approximately 5 cM apart from genomic region (129.77 cM) reported by [41]for plant height and the genomic region reported for tolerance index by [15]. A region significant for grain yield on chromosome 4A at 48.52 cM was close to locus Kukri_c12563_52 at 66.28 cM reported significant for effective number of spikes per square meter by [41]. The same study reported a genomic region on 5B at 39.64 cM for GY was 7 cM away from significant region for grain yield on 5B at 32.73 cM reported in the present study. Locus RAC875_c29431_1849 on 5B at 49.01 was close to genomic regions Tdurum_contig52439_196 at 40.56 cM [41], wsnp_CAP7_c2086_1018815 at 43.42 cM [55]and wsp_Ex_rep_c66651_64962429 at 49.01 cM [56] significantly associated with GY, normalized differential vegetative index at vegetative stage and thousand-kernel weight respectively. Locus BobWhite_c28058_232 and wsnp_Ex_c8400_14157060 at 134.06 cM and BS00076622_51 at on 148.65 cM on 7B were associated with yield in two out of four environments and similar regions were previously reported by [57] for SPN (134.5 cM) and KNL (149.5 cM).

Germplasm evaluation in multiple environmental conditions helps us to detect and compare QTLs for desired traits common among more than one condition. Genotype × environment interaction is the main problem in breeding for diverse adaptation in bread wheat. Occurrence of common genomic region among more than one stress shows common adaptation and tolerance mechanism of accessions under these stress treatments. In our study nine common regions were identified which were associated with traits under all four treatments i.e. [C], [D], [H] and [HD] treatment. In addition, two different stress indices were also used to find out stable regions across the environments. Multiple consistent regions for both stress indices were recorded on chromosome 2B and 5D. Many regions were shared among grain yield and stress indices for instance the genomic region on 5D at 67.24–71.76 cM was shared by DTM, GY, STI and TOL. A set of MTAs on chromosome 2B at 85.07 cM was near to IWB2285 locus at 70.4 cM significant for grain yield and grains per spike in collection of tetraploid wheat [54]. The genomic regions on 2B were present at 25.53, 27.64 and 37.03 cM, genomic regions in the same range (24.7 cM and 36.9 cM) as were reported for heat susceptibility index for kernel number, flag-leaf length and grain yield by [58]. The associations common among two or more treatments and associations significant for stress indices are of vital importance and can be used in marker-assisted selection to improve wheat tolerance to combined heat and drought stress.

Makers shared among more than one trait are very useful in marker-assisted selection as they may play a vital role in increasing QTL pyramiding efficiency. Multi trait markers were detected on all chromosomes in present study except 1D, 4D, 5A and 6B. These markers share two or more than two traits and maximum number of these multi trait markers was detected on 7B. The locus RAC875_rep_c110526_324 on 7B at 163.3 cM and chromosomal region on chromosome 7D at 208.1 cM were shared among 8 studied traits including GY. This implies that there are genetic bases for the high and consistent phenotypic correlation recorded among grain yield and these traits. The region on chromosome 7B was approximately 39 cM and 52 cM apart from two genomic regions M1441 and M7175 respectively identified by [59] for GY and associated traits in CIMMYT wheat accessions. The distal regions of chromosome 7A and 7B are reported to contain QTLs for grain yellow pigment content which is controlled by *Phytoene synthase* 1 (PSY-1) gene, presence of this gene might be responsible for pleiotropic associations on 7B [60]. Pleiotropic regions identified on chromosome 7D are may be due to pleiotropic effects of 7DL.7Ag translocation in wheat [61]. In addition to yield many genomic regions were shared between leaf characters (FLL, FLW, LA) and spike characters under stress treatments for example marker Excalibur_c26688_138 and BS00022775_51 on 1A and unmapped chromosome respectively were shared by FLW and GY. Another region on chromosome 7D at 202.54 cM was shared by FLL, PH, SL, TILL and BS00076622_51 on 7B at 148.65 cM was shared by FLW and HI. Sharing of these regions may be attributed to effective translocation of leaf photosynthates to growing spike and kernels during stress [62].

## Conclusion

The identification and introgression of major-effect QTLs are one of the best and proven approaches to improving the stress tolerance of wheat varieties. The accuracy and consistency of QTLs have clearly shown that structured association mapping with genome-wide molecular markers is an attractive option to identify major-effect QTLs for GY under different stress treatments. Association mapping results revealed many novel regions associated with combined drought and heat stress tolerance, along with nine consistent regions common among all four treatments and many pleiotropic regions associated with more than one traits. Further exploration of common regions among treatments through marker assisted selection can help in understanding complex mechanisms of abiotic stress tolerance and fine mapping of these regions can lead to new gene discoveries. Intensity of climate change will increase in next few years and will decrease wheat production, thus it is need of time to improve germplasm by arranging crosses between diverse parents and improve genomic technology and couple these technologies with marker assisted selection. The present study will help in understanding irrigation use efficiency and tolerance of exotic lines to rain fed conditions. It will help in identification and inclusion of tolerant genotypes in breeding program for further strengthening.

## Supporting information

S1 Table Passport data about section history, entry number and population structure of germplasm used in the present study. (XLSX)

S2 Table GWAS results for yield, yield related traits and stress indices calculated from BLUEs values obtained from two-year data for four different stress treatments. (XLSX)

S1 Fig Number of days for stress treatments and percent moisture content maintained during stress treatments. (TIF)

S2 Fig Chromosome wise average major allele frequency, genetic diversity and polymorphic information content of all polymorphic markers. (TIF)

S1 Appendix Chromosome wise average linkage disequilibrium and correlation among all polymorphic markers. (PDF)

S2 Appendix Distributions of mean values across treatment, Manhattan and QQ plots for yield traits and stress indices. (PDF)

## Abbreviations

DW/Biomass: Plant above ground dry weight
DTM: Days to maturity
FDR: False discovery rate
FLL: Flag leaf length
FLW: Flag leaf width
GWAS: Genome wide association studies
GY: grain yield, HI: Harvest index
LA: Leaf area
LD: Linkage disequilibrium
MAF: Minor allele frequency
MTAs: Marker trait associations
PH: Plant height
SL: Spike length
SPLS: Spikelets per spike
TILL: Tillers per plant
STI: Stress tolerance index
TOL: Tolerance index
