# Supplementary figures and images for: Genome-wide association analyses for yield and yield-related traits in bread wheat (Triticum aestivum L.) under pre-anthesis combined heat and drought stress in field conditions
Source: PLoS One. 2019 Mar 18;14(3):e0213407. doi: 10.1371/journal.pone.0213407 (PMC6422278; doi:10.1371/journal.pone.0213407)

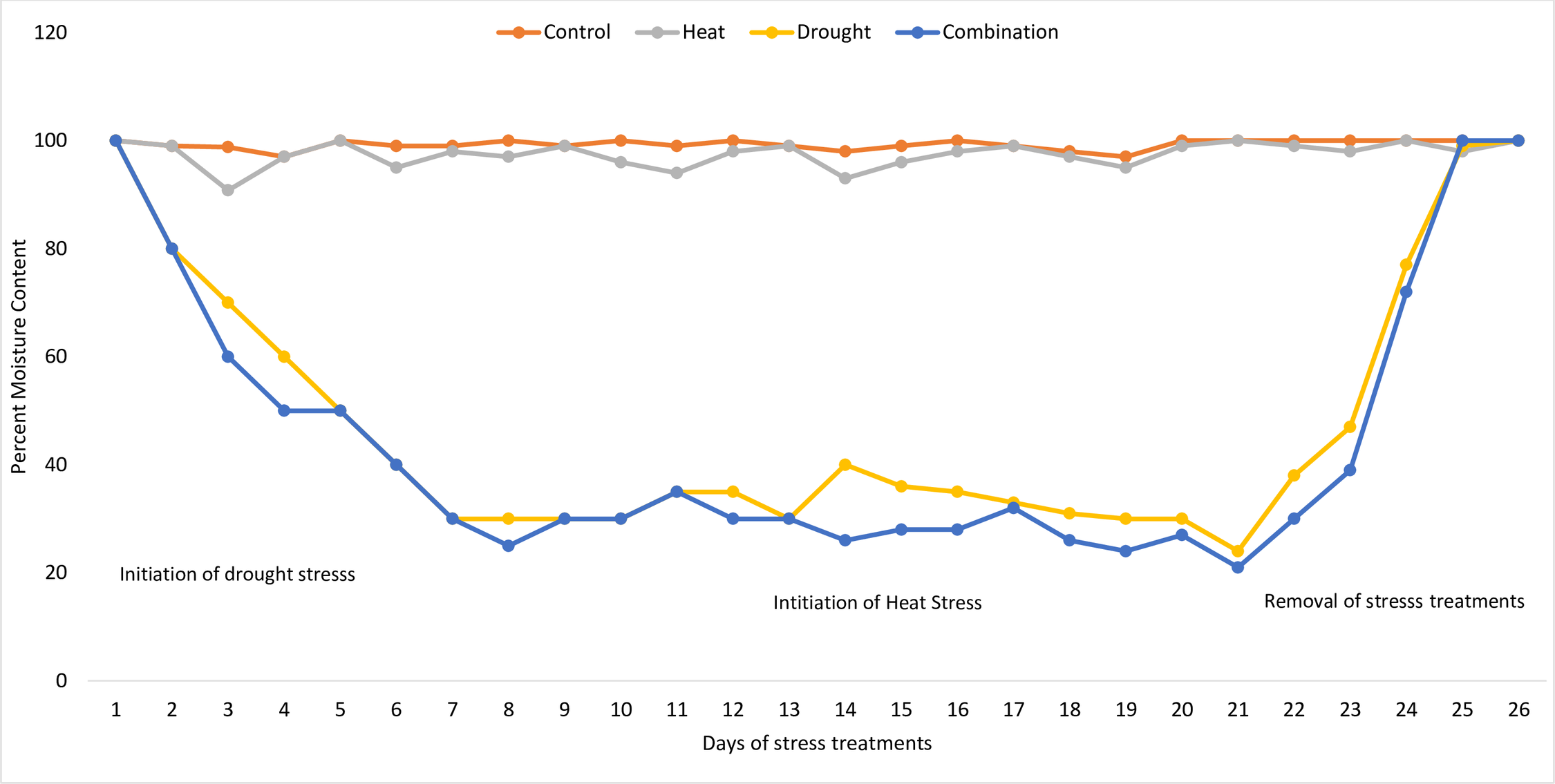

Supplement: S1 Fig — (TIF) [file pone.0213407.s003.tif]

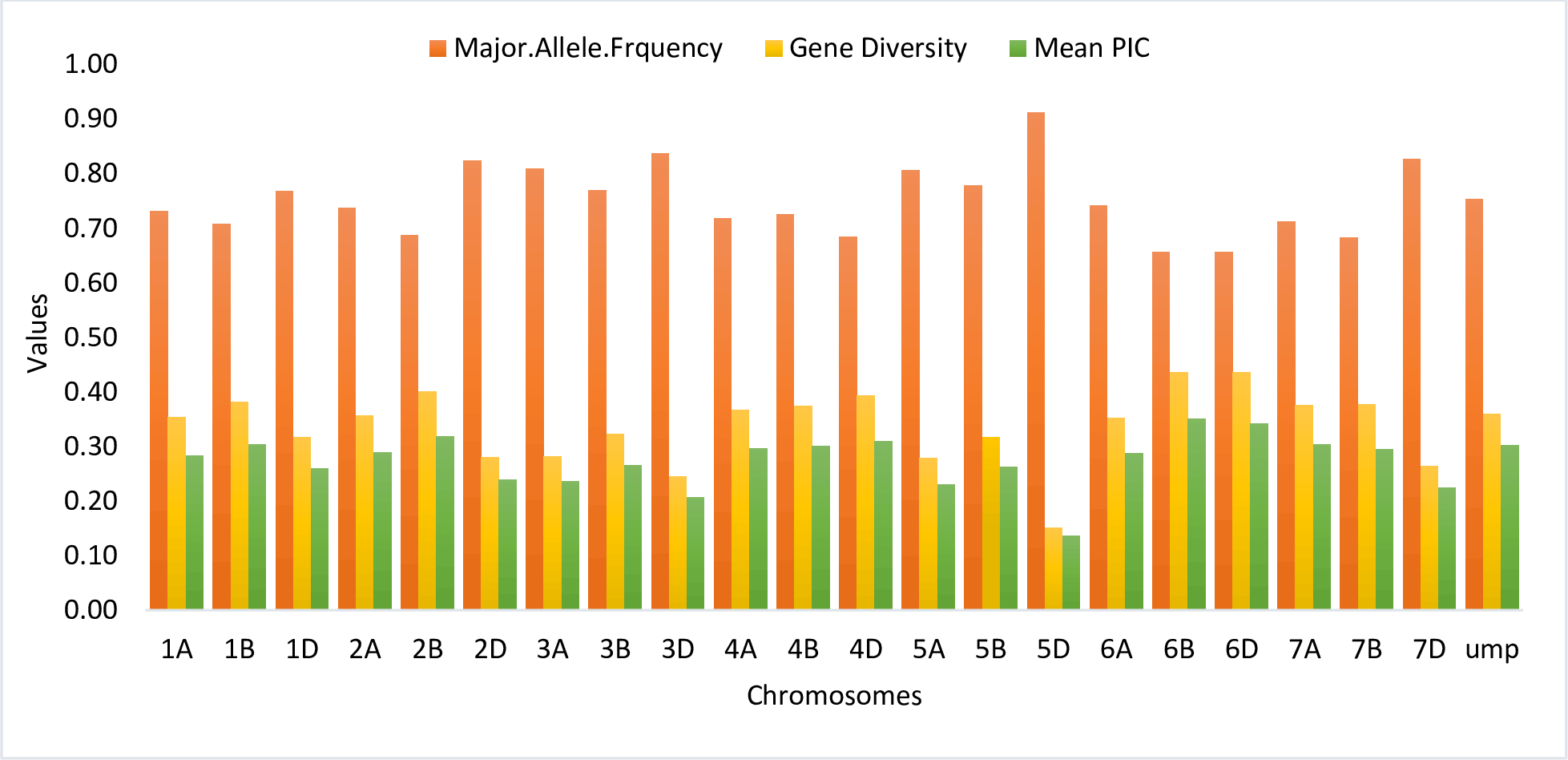

Supplement: S2 Fig — (TIF) [file pone.0213407.s004.tif]
