# Supplementary material for: Genome-wide association analyses for yield and yield-related traits in bread wheat (Triticum aestivum L.) under pre-anthesis combined heat and drought stress in field conditions
Source: PLoS One. 2019 Mar 18;14(3):e0213407. doi: 10.1371/journal.pone.0213407 (PMC6422278; doi:10.1371/journal.pone.0213407)
Supplement: S2 Appendix — (PDF) [file pone.0213407.s006.pdf]

GWAS RESULTS

Distributions, Manhattan and QQ plots for Yield Traits and Stress Indices

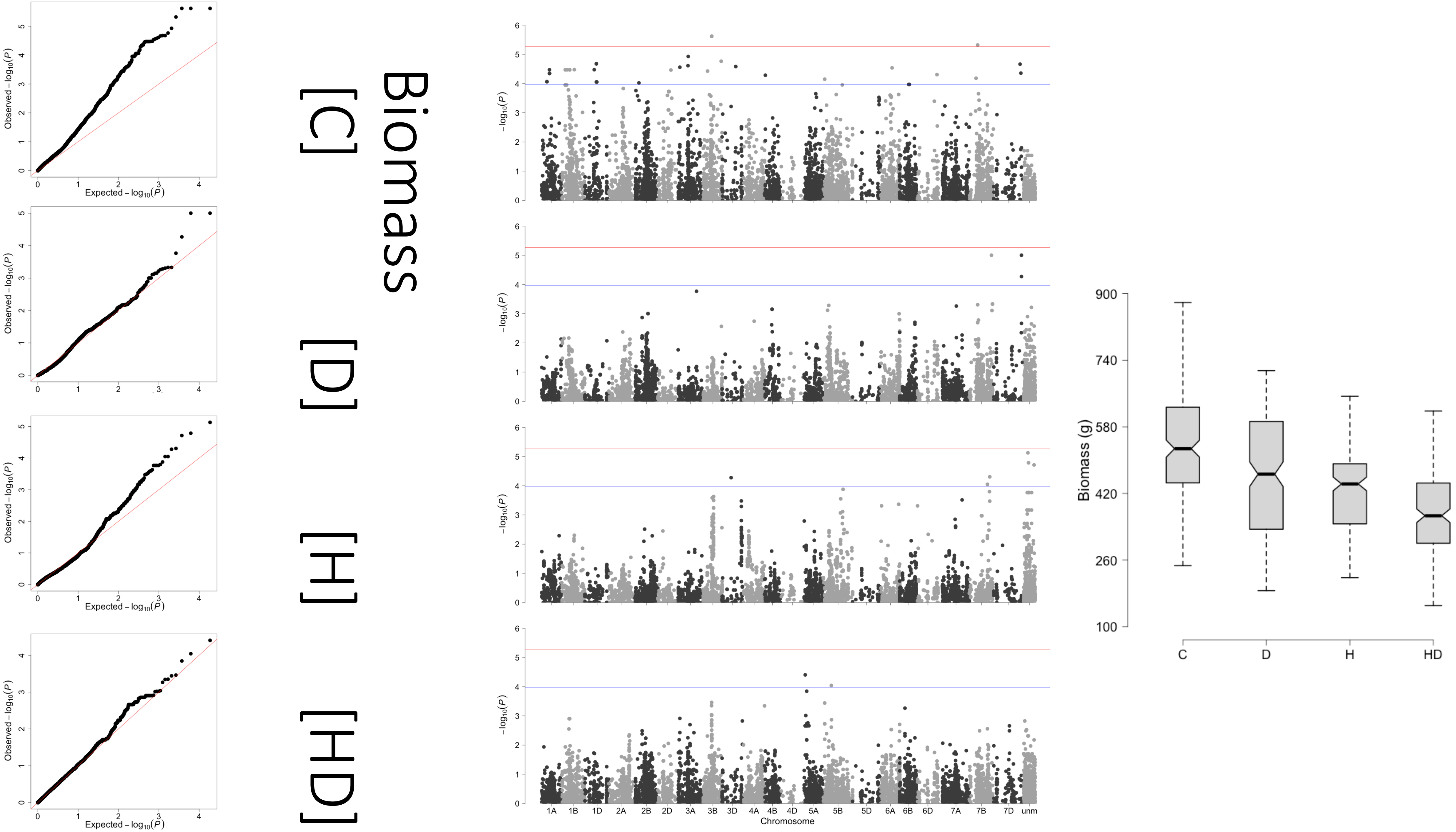

Biomass
[C]
[D]
[H]
[HD]
Observed − log10(P)
Expected − log10(P)
−log10(P)
1A 1B 1D 2A 2B 2D 3A 3B 3D 4A 4B 4D 5A 5B 5D 6A 6B 6D 7A 7B 7D unm
Chromosome
Biomass (g)
900
740
580
420
260
100
C
D
H
HD

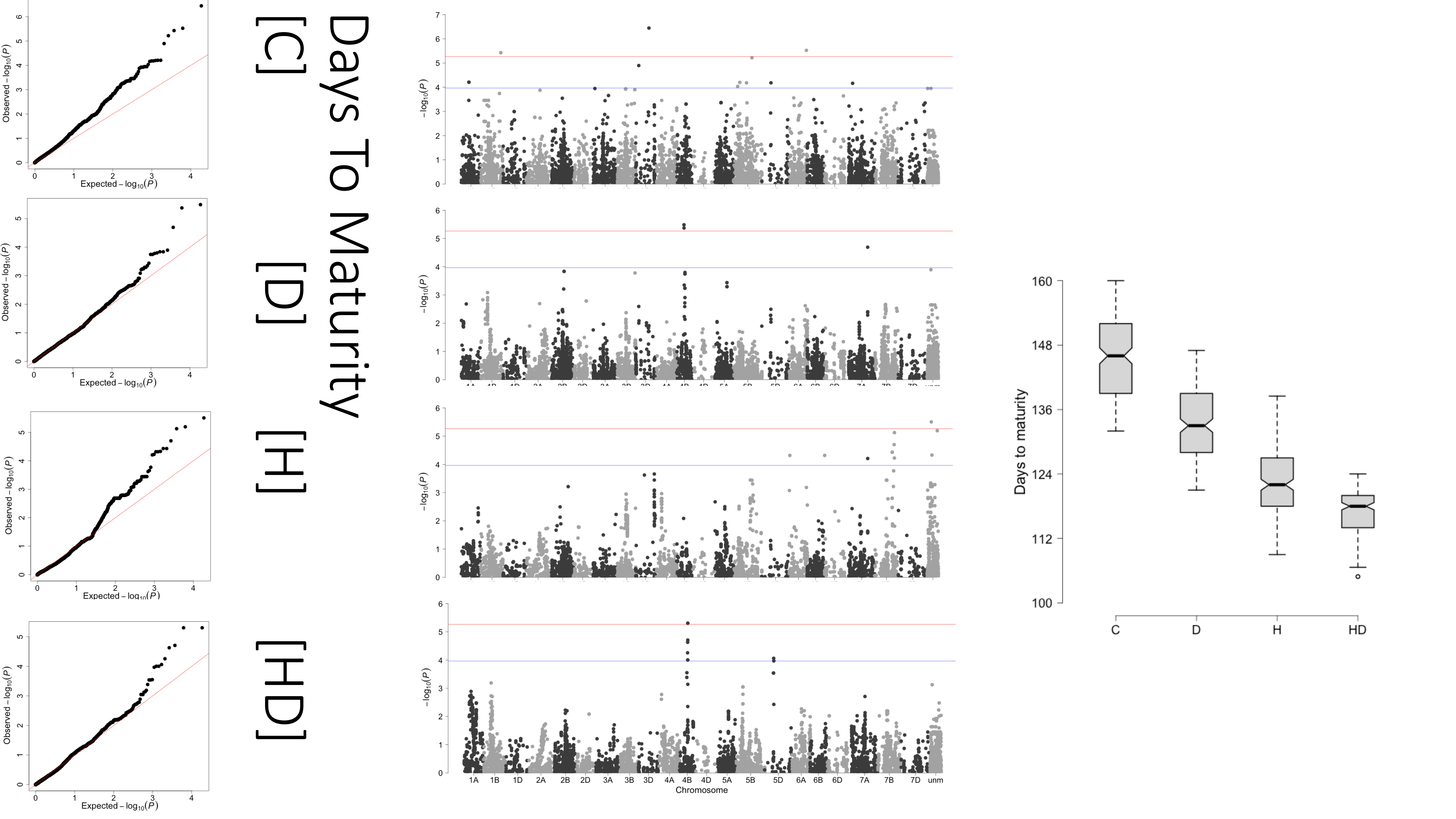

Days To Maturity
[C]
[D]
[H]
[HD]
Observed − log10(P)
Expected − log10(P)
−log10(P)
Chromosome
1A 1B 1D 2A 2B 2D 3A 3B 3D 4A 4B 4D 5A 5B 5D 6A 6B 6D 7A 7B 7D unm
Days to maturity
160
148
136
124
112
100
C
D
H
HD

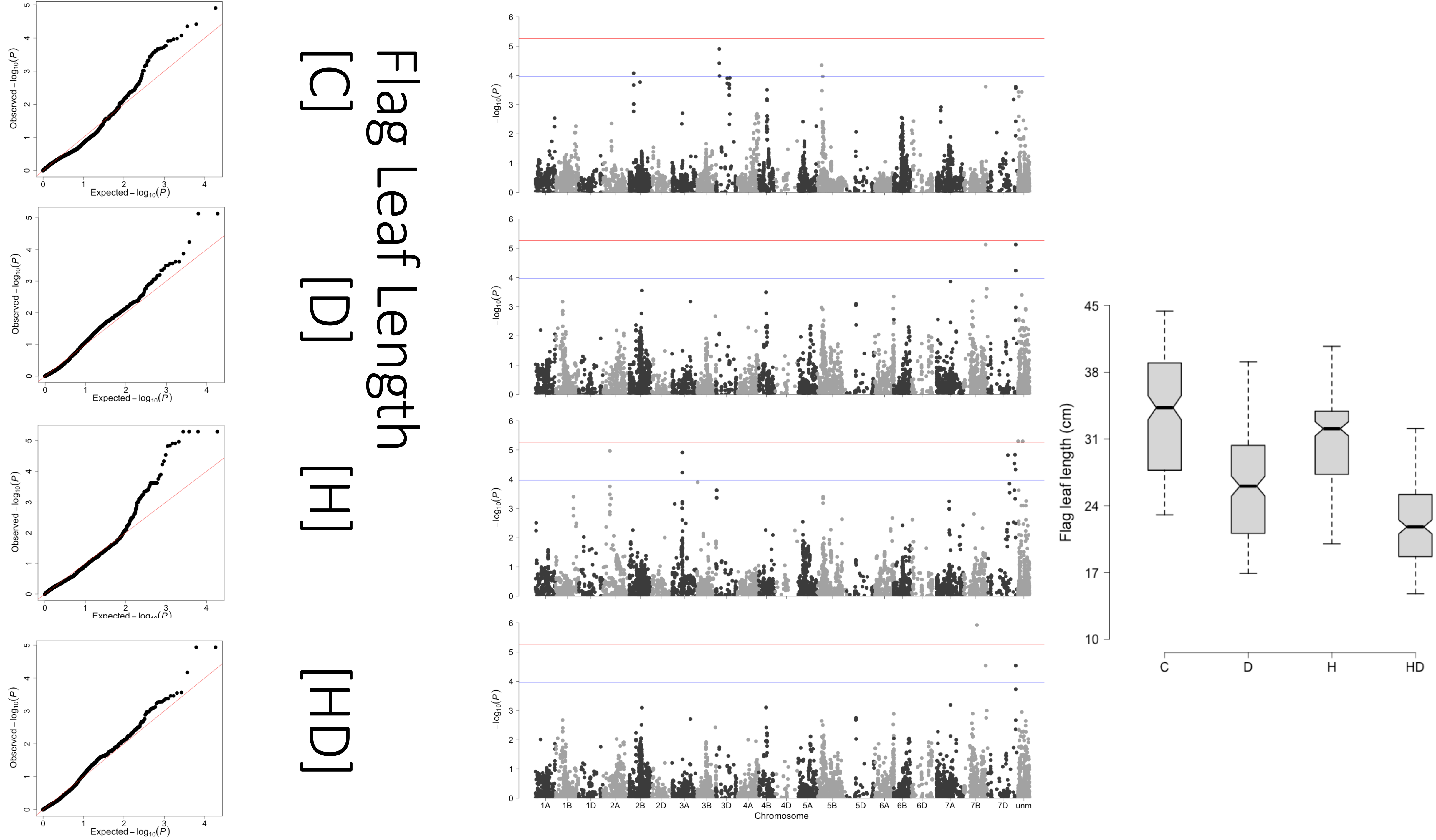
Flag Leaf Length
[C]
[D]
[H]
[HD]
Observed − log10(P)
Expected − log10(P)
−log10(P)
1A
1B
1D
2A
2B
2D
3A
3B
3D
4A
4B
4D
5A
5B
5D
6A
6B
6D
7A
7B
7D
unm
Chromosome
Flag leaf length (cm)
45
38
31
24
17
10
C
D
H
HD

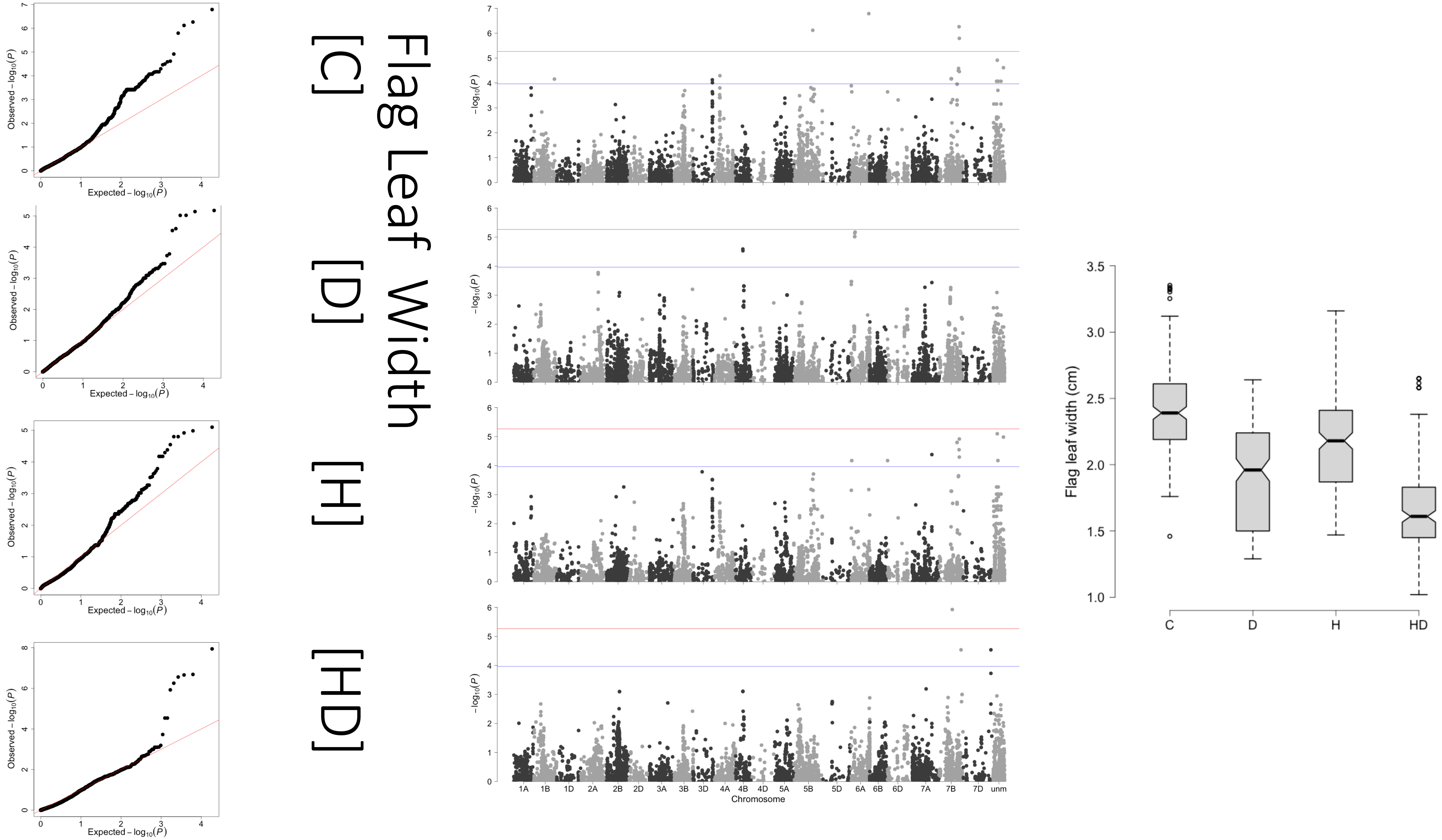
Flag Leaf Width
[C]
[D]
[H]
[HD]
Expected − log10(P)
Observed − log10(P)
−log10(P)
1A 1B 1D 2A 2B 2D 3A 3B 3D 4A 4B 4D 5A 5B 5D 6A 6B 6D 7A 7B 7D unm
Chromosome
Flag leaf width (cm)
C
D
H
HD

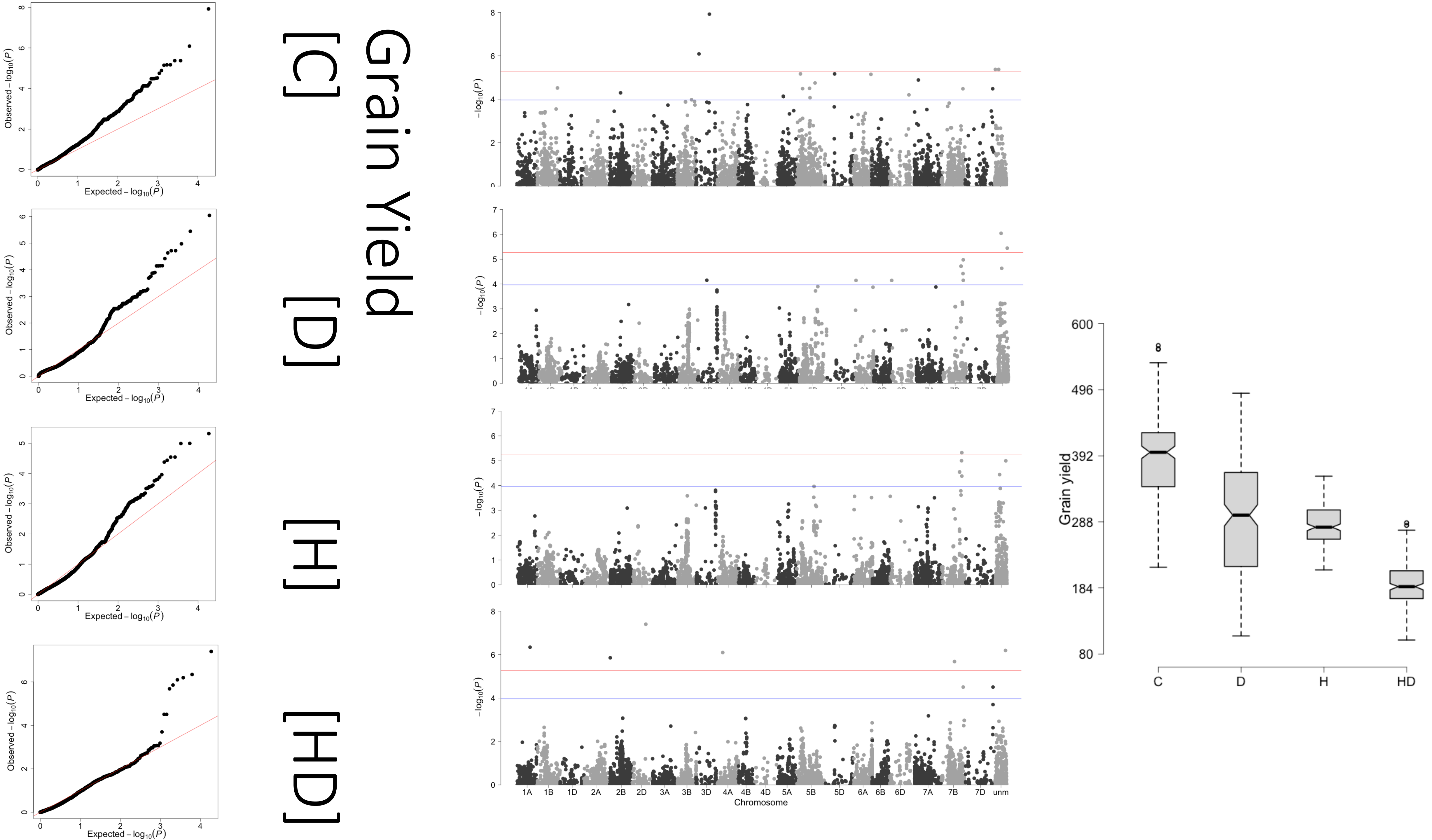
Grain Yield
[C]
[D]
[H]
[HD]
Observed – log10(P)
Expected – log10(P)
–log10(P)
1A 1B 1D 2A 2B 2D 3A 3B 3D 4A 4B 4D 5A 5B 5D 6A 6B 6D 7A 7B 7D unm
Chromosome
Grain yield
600
496
392
288
184
80
C
D
H
HD

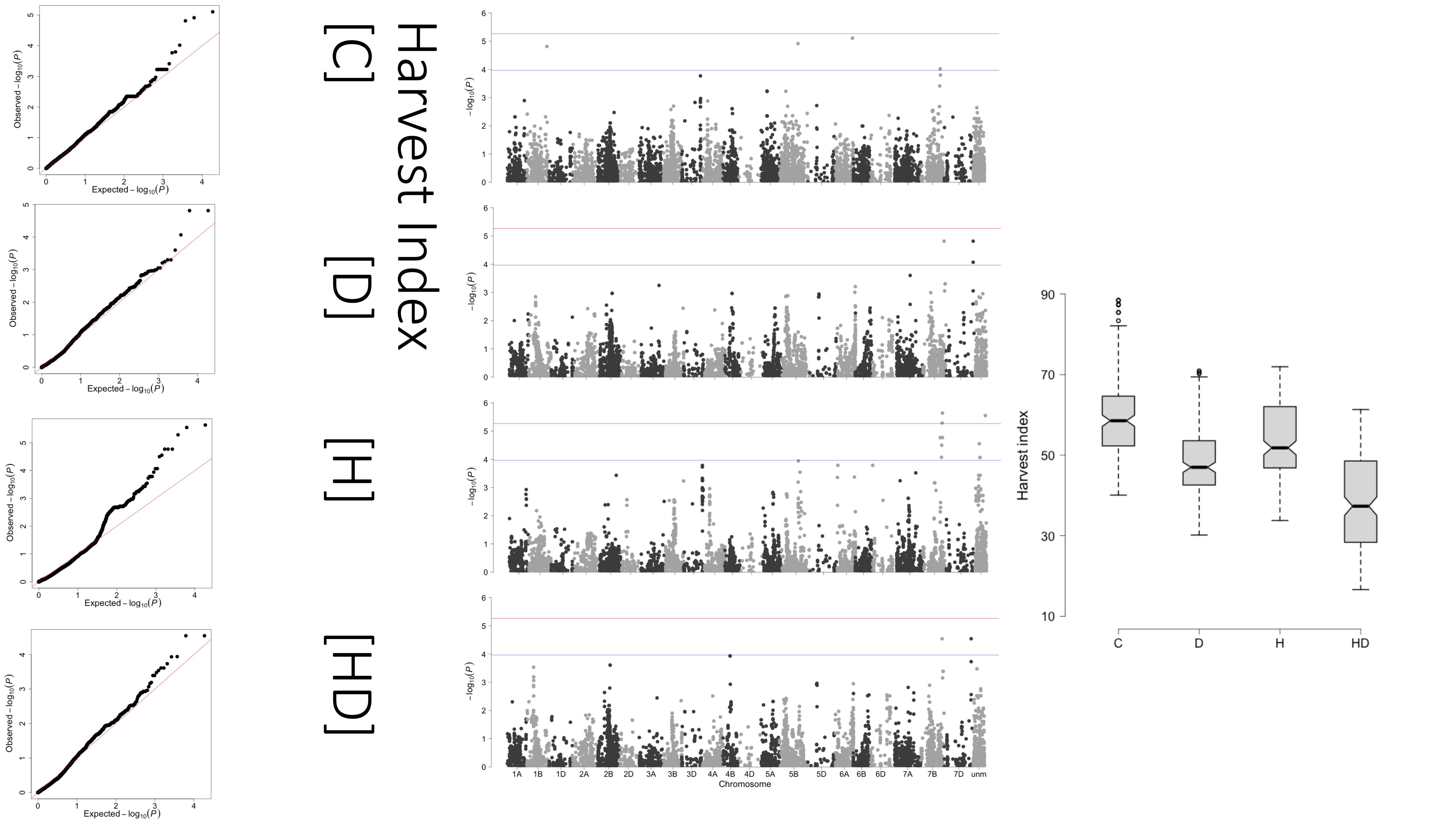
Harvest Index
[C]
[D]
[H]
[HD]
Observed − log10(P)
Expected − log10(P)
−log10(P)
1A
1B
1D
2A
2B
2D
3A
3B
3D
4A
4B
4D
5A
5B
5D
6A
6B
6D
7A
7B
7D
unm
Chromosome
Harvest index
C
D
H
HD

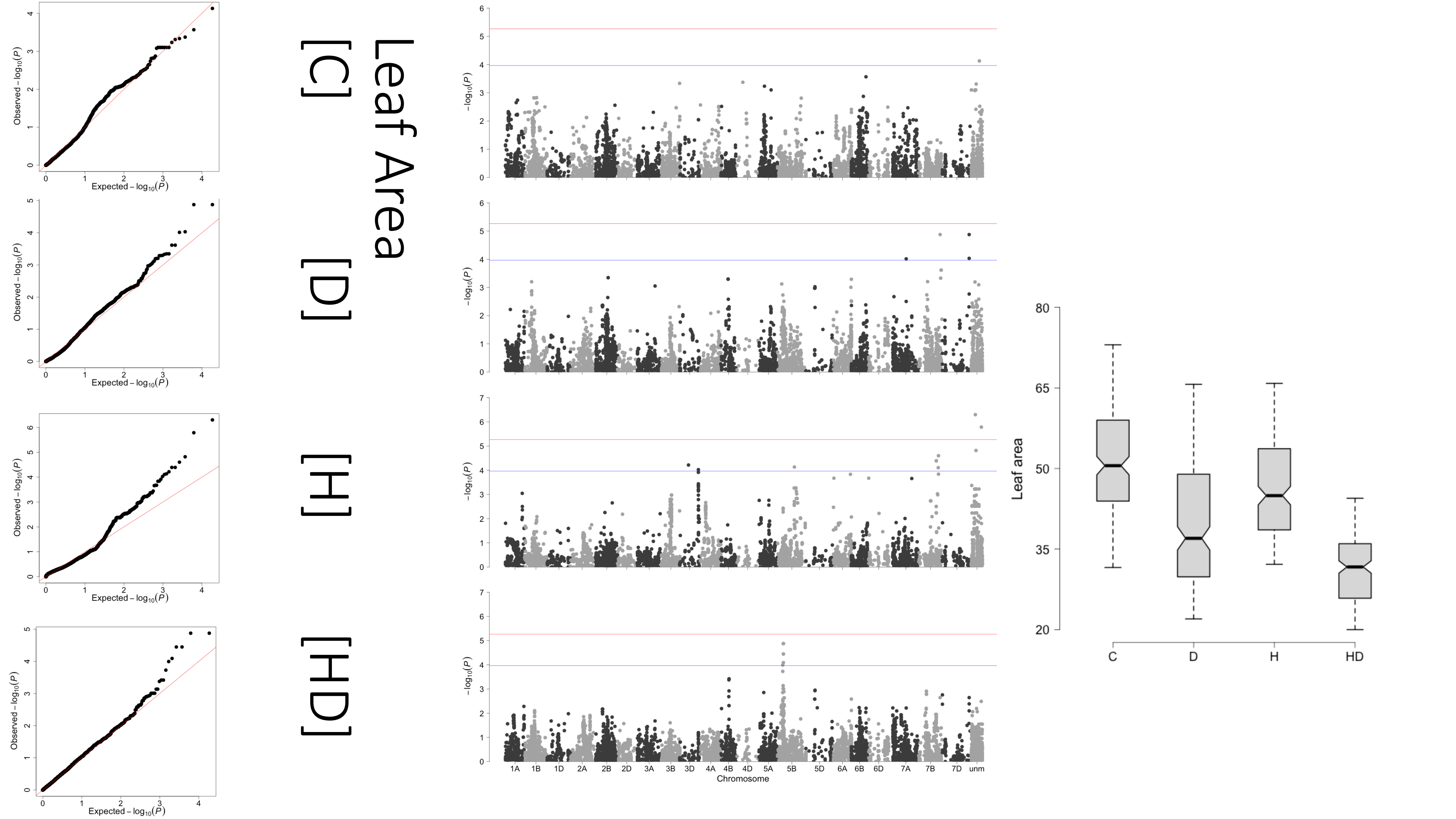

Leaf Area
[C]
[D]
[H]
[HD]
Observed − log10(P)
Expected − log10(P)
−log10(P)
1A 1B 1D 2A 2B 2D 3A 3B 3D 4A 4B 4D 5A 5B 5D 6A 6B 6D 7A 7B 7D unm
Chromosome
Leaf area
C
D
H
HD

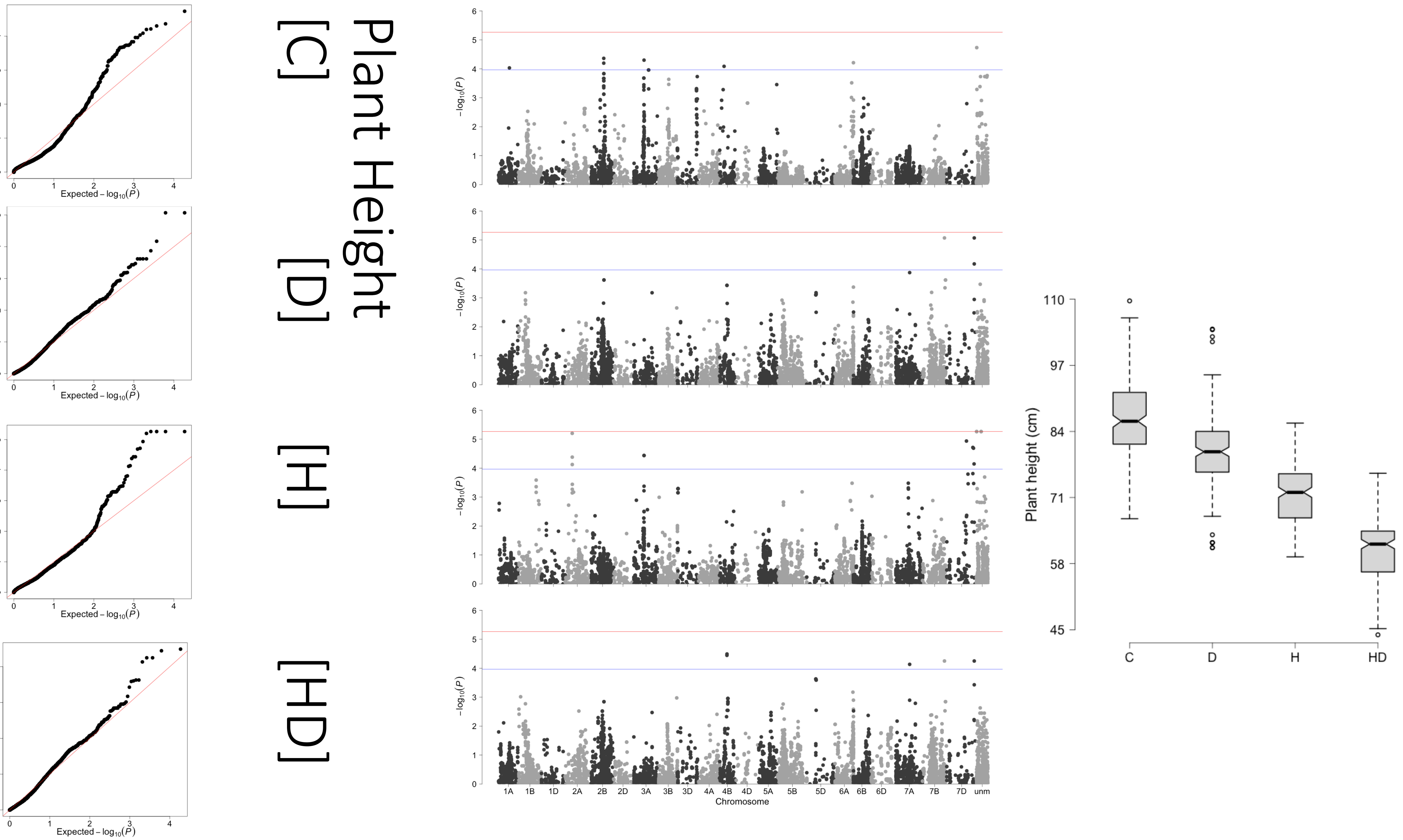

Plant Height
[C]
[D]
[H]
[HD]
Expected – log10(P)
−log10(P)
1A 1B 1D 2A 2B 2D 3A 3B 3D 4A 4B 4D 5A 5B 5D 6A 6B 6D 7A 7B 7D unm
Chromosome
Plant height (cm)
110
97
84
71
58
45
C
D
H
HD

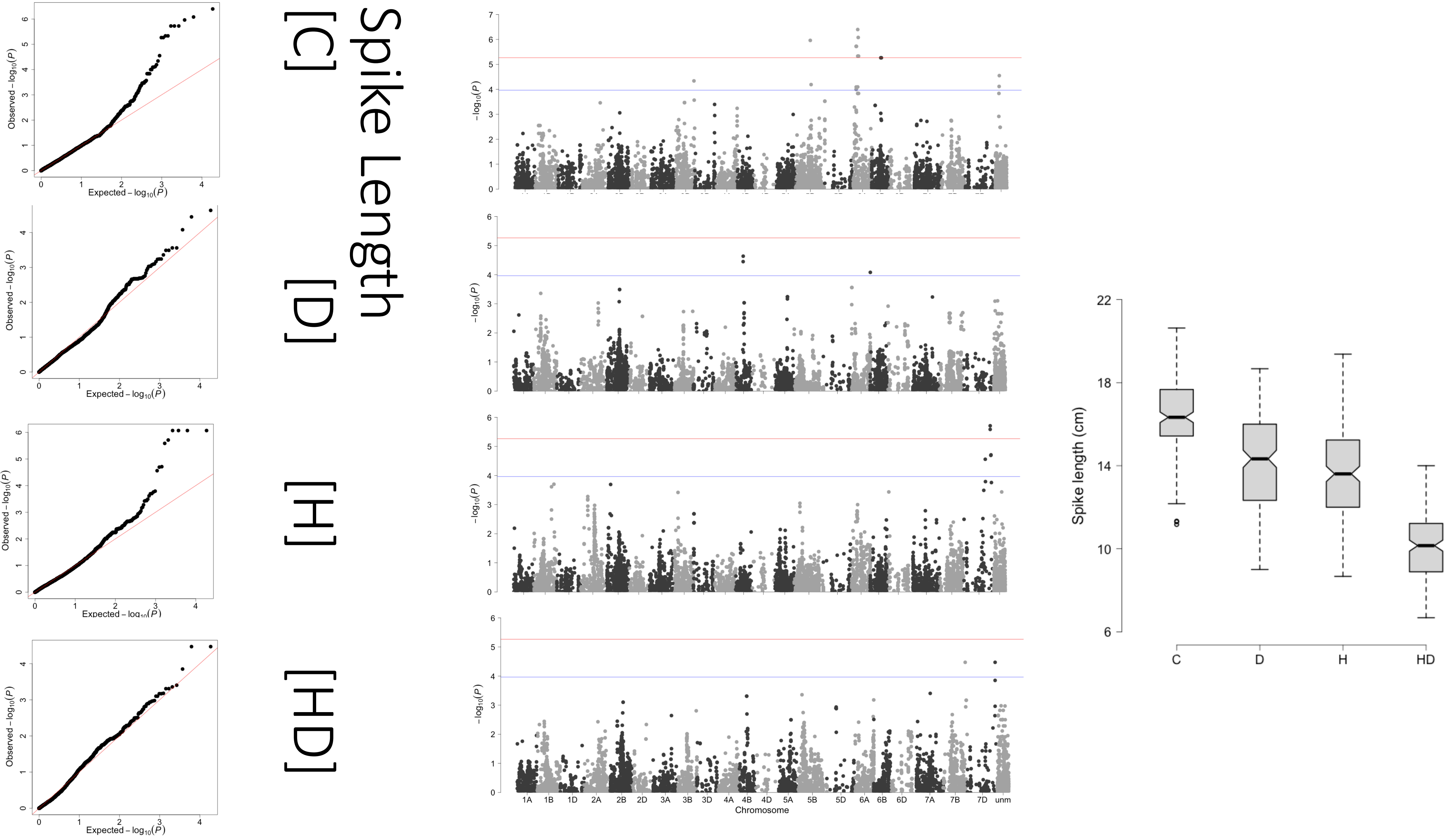
Spike Length
[C]
[D]
[H]
[HD]
Observed − $\log_{10}(P)$
Expected − $\log_{10}(P)$
$-\log_{10}(P)$
1A 1B 1D 2A 2B 2D 3A 3B 3D 4A 4B 4D 5A 5B 5D 6A 6B 6D 7A 7B 7D unm
Chromosome
Spike length (cm)
C
D
H
HD

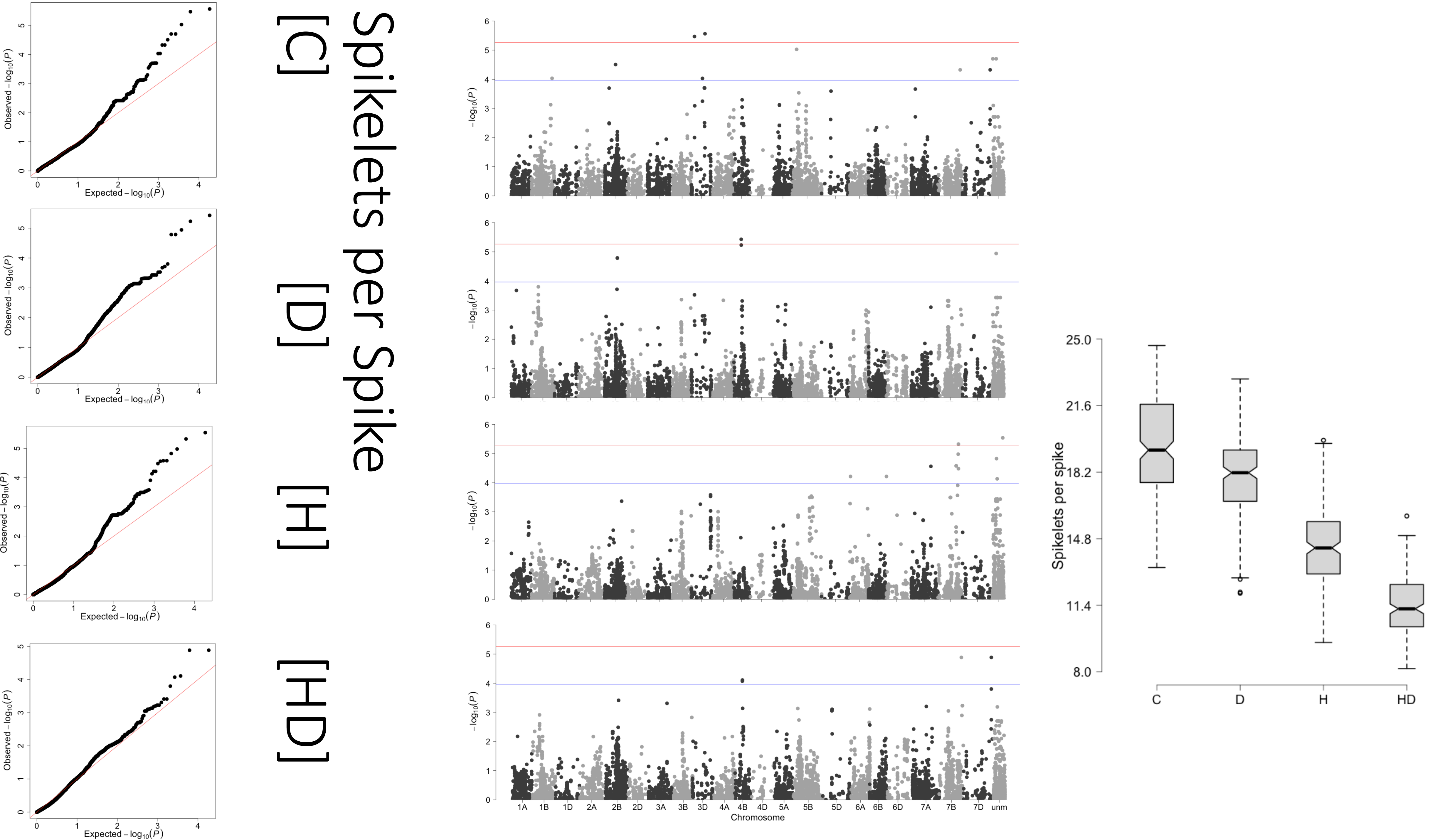
Spikelets per Spike
[C]
[D]
[H]
[HD]
Observed − log10(P)
Expected − log10(P)
−log10(P)
1A 1B 1D 2A 2B 2D 3A 3B 3D 4A 4B 4D 5A 5B 5D 6A 6B 6D 7A 7B 7D unm
Chromosome
Spikelets per spike
25.0
21.6
18.2
14.8
11.4
8.0
C
D
H
HD

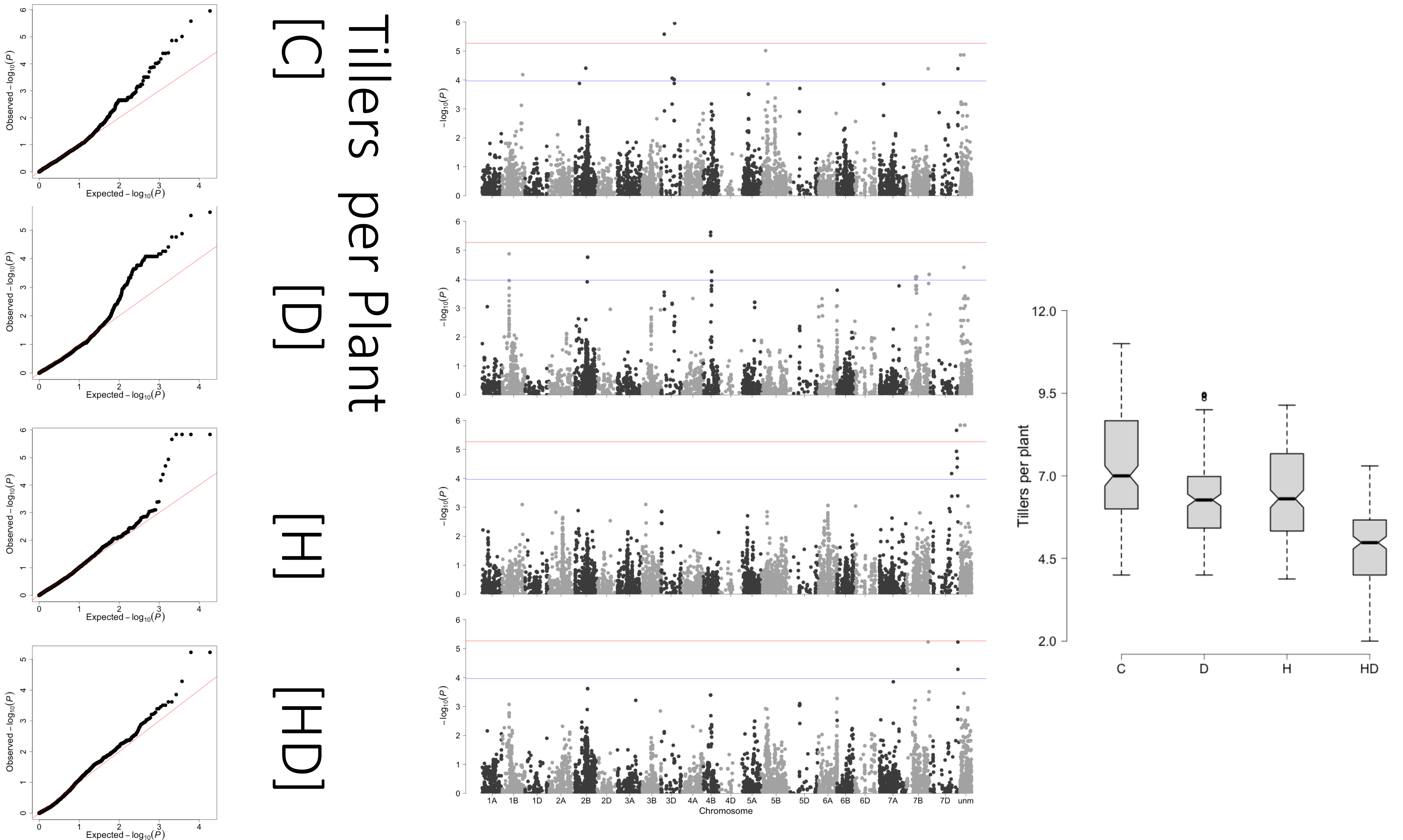

Tillers per Plant
[C]
[D]
[H]
[HD]
Observed − log10(P)
Expected − log10(P)
−log10(P)
1A 1B 1D 2A 2B 2D 3A 3B 3D 4A 4B 4D 5A 5B 5D 6A 6B 6D 7A 7B 7D unm
Chromosome
Tillers per plant
12.0
9.5
7.0
4.5
2.0
C
D
H
HD

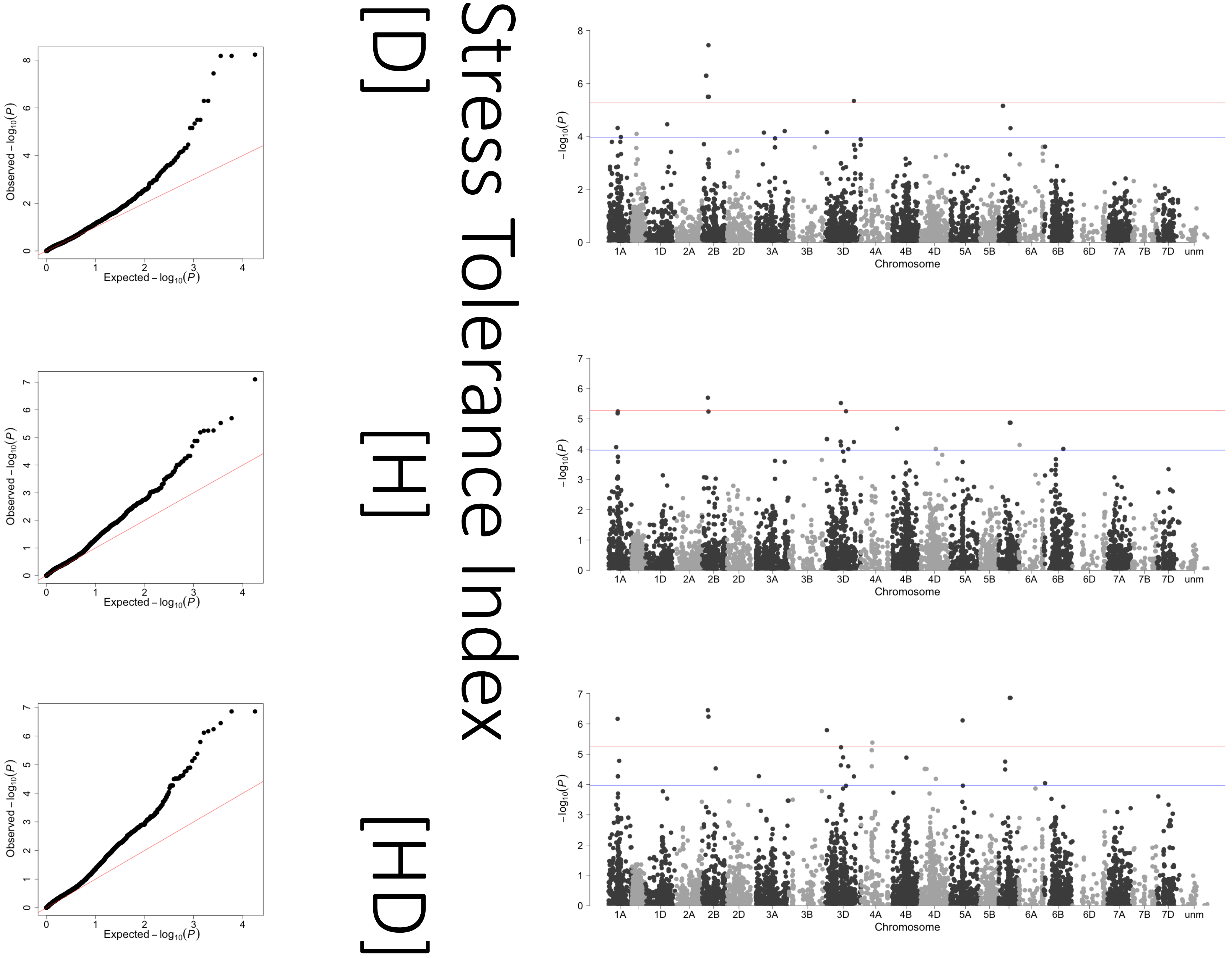
Stress Tolerance Index
[D]
[H]
[HD]
Observed − log10(P)
Expected − log10(P)
−log10(P)
Chromosome
1A 1D 2A 2B 2D 3A 3B 3D 4A 4B 4D 5A 5B 6A 6B 6D 7A 7B 7D unm

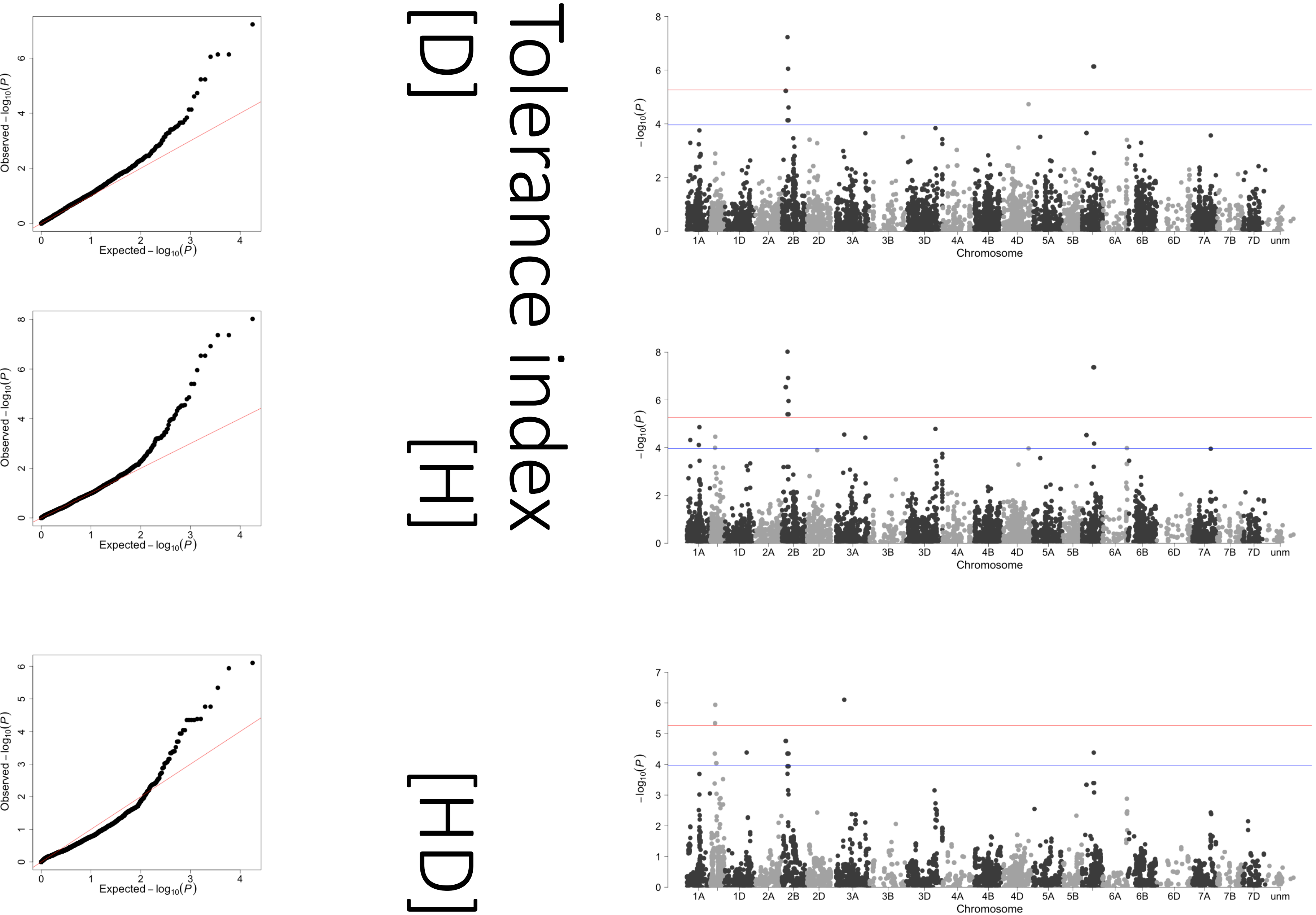
Tolerance index
[D]
[H]
[HD]
Observed − log10(P)
Expected − log10(P)
−log10(P)
1A 1D 2A 2B 2D 3A 3B 3D 4A 4B 4D 5A 5B 6A 6B 6D 7A 7B 7D unm
Chromosome
